# Comparative efficacy of aripiprazole and paliperidone to treatment-as-usual in managing stimulant-induced psychosis: a 24-month randomized controlled trial

**DOI:** 10.3389/fphar.2026.1892182

**Published:** 2026-08-12

**Authors:** Albert Kar Kin Chung, Sau Wan Tang, Cheuk Yin Tse, Johnson Kai Chun Law, Chi Kei Lee, Welton Leung, Y. Doug Dong, Fu Chan

**Affiliations:** 1 Department of Psychiatry, Li Ka Shing Faculty of Medicine, The University of Hong Kong, Pokfulam, Hong Kong SAR, China; 2 Department of Psychiatry, North District Hospital, Hospital Authority, Hong Kong, Hong Kong SAR, China

**Keywords:** antipsychotic, efficacy, randomized controlled trial, schizophrenia, stimulant use disorder, stimulant-induced psychosis

## Abstract

**Objectives:**

Stimulant use can induce psychotic symptoms and, in some cases, lead to persistent psychotic disorders and schizophrenia. Whether early antipsychotic treatment improves outcomes in stimulant-induced psychosis (StIP) remains unclear.

**Trial Design:**

This prospective 24-month, single-blind, three-arm randomized controlled trial evaluated the efficacy of aripiprazole and paliperidone compared with treatment-as-usual (TAU) in adults with StIP.

**Methods:**

Between June 2019 and May 2022, 165 adults (mean age 38.69 [SD 10.08]) meeting DSM-5 criteria with cocaine- or methamphetamine-related StIP were randomized (1:1:2) to aripiprazole, paliperidone, or TAU using the sealed envelope method. The primary outcomes were changes in Clinical Global Impression-Severity (CGI-S), CGI-Improvement (CGI-I), and CGI-Efficacy index (CGI-E) at 12 months (primary endpoint) and 24 months (secondary endpoint), analyzed using mixed models for repeated measures in the intention-to-treat sample. Secondary outcomes included psychotic symptom severity, stimulant use, cognitive function, and psychosocial functioning. Assessors were blinded during the first 12 months then unblinded during 12–24 months.

**Results:**

Aripiprazole showed significantly greater reductions in CGI-S than TAU at 12 months (*p* = 0.03, Cohen’s *d* = −0.31) that was sustained at 24 months (*p* = 0.03, *d* = −0.38). Paliperidone did not differ significantly from TAU on CGI-S at either timepoint. Both aripiprazole and paliperidone improved CGI-I and CGI-E at 12 months, but only aripiprazole maintained superiority over TAU at 24 months (aripiprazole at 24 months: both *p* < 0.05, CGI-I: *d* = −0.40, CGI-E: *d* = −1.71). Four participants experienced serious adverse events. GASS measured side-effects were generally mild across all groups. Exploratory analyses suggested possible cognitive worsening and higher rates of stimulant-positive urine tests with paliperidone at 24 months. The overall 24-month conversion rate from StIP to schizophrenia was 5.11% and did not differ between groups.

**Conclusion:**

Aripiprazole demonstrated consistent favorable CGI-based outcome responses and was well-tolerated in managing StIP compared with TAU over 24 months, whereas paliperidone showed more limited long-term benefits. Large placebo-controlled trials are warranted to determine if early assertive antipsychotic interventions causally reduce the risk of progression from StIP to schizophrenia.

**Clinical Trial Registration:**

https://clinicaltrials.gov/study/NCT03485417, NCT03485417.

## 1 Introduction

Cocaine and methamphetamine are the two most commonly misused stimulants among treatment-seeking individuals who use drugs (UNODC, 2024). Their misuse is associated with a high prevalence of stimulant-induced psychosis (StIP), reported to reach 40% for cocaine and 60% for methamphetamine (Boden et al., 2023; Sabe et al., 2021). Moreover, stimulant misuse substantially elevates the risks of developing chronic psychotic disorders and schizophrenia (Bouthillier et al., 2026; Murrie et al., 2020; Niemi-Pynttäri et al., 2013).

Cocaine and methamphetamine act as indirect dopamine agonists with potent effects on the mesolimbic dopamine pathway, underlying their high misuse potential and substantial capacity to induce psychotic symptoms (Wang et al., 2025). Repeated stimulant exposure can also produce “kindling” effects that further lead to long-term sensitization of dopaminergic release in the striatum, thereby predisposing individuals who use stimulants to high vulnerability to StIP and schizophrenia (Vanderschuren et al., 1999; Vezina, 2007). Although cocaine and methamphetamine differ in their precise mechanisms of action and pharmacokinetics, the severity of the related psychosis and the associated neurocognitive declines do not differ significantly between individuals with cocaine and/or methamphetamine dependence (Alexander et al., 2017). Compared with individuals diagnosed with schizophrenia, however, both stimulant-dependent groups exhibit significantly less severe psychosis (Alexander et al., 2017; Alexander et al., 2019).

Short-term (three-to six-week) randomized trials have demonstrated that olanzapine and quetiapine are superior to risperidone, paliperidone or aripiprazole, but not to haloperidol, in managing methamphetamine-induced psychosis (Srisurapanont et al., 2021). Low-dose antipsychotic treatment in StIP also appears beneficial in averting the likelihood of progression to chronic psychosis and schizophrenia (Curran et al., 2004). Nevertheless, traditional antipsychotics with potent dopamine D2 affinity may exacerbate compulsive drug-seeking and drug-taking behaviors by blocking D2 receptors in the limbic striatum and thereby disrupting with reward circuits. In contrast, second-generation antipsychotics, whose primary actions involve balanced D2/D3 and serotonin 5HT2A antagonisms, carry a lower risk of such interference (Lalanne et al., 2016; Samaha, 2014). Open-label studies of low dose risperidone have shown to reduce methamphetamine use among dependent individuals (Karila et al., 2010; Meredith et al., 2009). Aripiprazole, a partial D2 agonist, has been shown to attenuate the discriminative stimulus of d-amphetamine in healthy volunteers (Stoops, 2006), to diminish the reinforcing effects of oral methamphetamine in recreational users (Stoops et al., 2013), and to reduce the self-reported effects of cocaine in cocaine-dependent individuals (Lile et al., 2011). However, fixed-dose oral aripiprazole failed to demonstrate its effectiveness in clinical trials for either intravenous amphetamine (Tiihonen et al., 2007) or methamphetamine (Newton et al., 2008) dependence. Evidence regarding paliperidone on stimulant dependence remains limited (Wang et al., 2019; 2020), yet its minimal hepatic metabolism offers a potential safety advantage over other antipsychotics in the context of stimulant-related hepatotoxicity when managing StIP (Janssen, 2025; Robinson et al., 2024).

Individuals with co-occurring diagnoses of substance dependence, psychosis and schizophrenia spectrum disorders are prone to premature treatment discontinuation and poor adherence to oral medications (Colizzi et al., 2016; Engh and Bramness, 2017; Kane et al., 2013). Stimulant users who experience StIP similarly show higher drop-out rates from psychiatric services (Crebbin et al., 2009). Early assertive antipsychotic intervention is therefore crucial to limit the progression of StIP to schizophrenia (Lappin et al., 2017). At present, robust evidence is still lacking regarding the best option of antipsychotic medications and their long-term efficacy in treating StIP. Their effects on stimulant dependence also remain inconclusive (Martinotti et al., 2022).

To address these gaps, we conducted a 24-month randomized controlled trial (RCT) to investigate whether early assertive antipsychotic intervention using aripiprazole or paliperidone, compared with treatment-as-usual (TAU), would improve psychosis outcomes in individuals with StIP, reduce their stimulant dependence, and lower the rate of conversion from StIP to schizophrenia.

## 2 Materials and methods

### 2.1 Study design and participants

The present study was a 24-month, two-phase, three-arm longitudinal RCT comprising two consecutive 12-month phases. In the first single-blind Active Intervention phase, participants were randomized to receive aripiprazole, paliperidone, or TAU for 12 months. In the second open-label Observation Maintenance phase, participants in all three groups could choose to continue or discontinue their assigned treatment from the Active Intervention phase for an additional 12 months.

Participants were recruited via random sampling from community, specialized substance misuse clinics, and general psychiatric and medical wards and clinics in Hong Kong. In brief, individuals aged 16–50 years who were using stimulants as their primary drug and suffering stimulant induced psychotic symptoms were eligible for recruitment. Detailed inclusion and exclusion criteria are provided in Supplementary Table S1. All participants provided written informed consent prior to any study procedures. They would receive USD 25 as honorarium upon completion of each follow-up assessment.

This study was approved by the Institutional Review Board of the University of Hong Kong/Hospital Authority Hong Kong West Cluster (IRB reference: UW 18–094) and the Joint Chinese University of Hong Kong-New Territories East Cluster Clinical Research Ethics Committee (CREC reference: 2018.156-T). This RCT also obtained the Certificates of Clinical Trial from the Department of Health, HKSAR (No.101536, No.101128, and No.200061) and was registered at the ClinicalTrials.gov (NCT03485417). All study procedures were conducted in accordance with the Declaration of Helsinki.

### 2.2 Randomization and blinding

Consented participants were randomly assigned in a 1:1:2 ratio to aripiprazole, paliperidone or TAU using the sealed envelope method. An independent staff member not involved in the study drew an opaque envelope containing the treatment allocation from an opaque box and handed it to the attending psychiatrist, who then prescribed the assigned treatment.

During the initial 12-month Active Intervention phase, a single-blinding design was employed. Independent assessors who conducted all the semi-structured interviews and outcome rating scales were blinded to treatment allocation. Attending psychiatrists who prescribed the study medications did not perform outcome assessments on the participants they treated. Participants were explicitly instructed not to reveal any details of their assigned treatment to the assessors to protect blinding. In the subsequent 12-month Observation Maintenance phase, all assessors were unblinded. Attending psychiatrists who administered the randomized interventions during the Active Intervention phase then were permitted to perform outcome assessments on participants they had previously treated.

### 2.3 Medications

**FIGURE 1:**
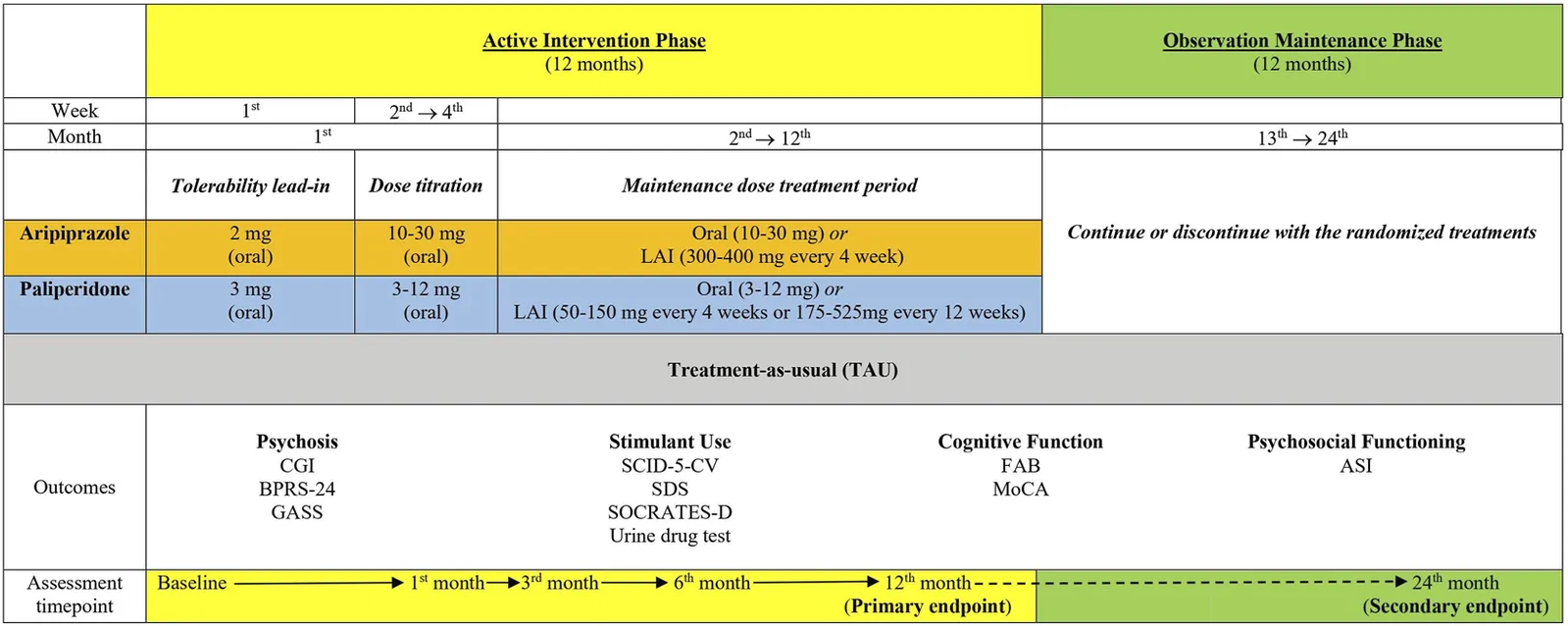
Schedules of treatment administration and follow-up assessment timepoints for the three treatment groups over the 24-month study period.

Following randomization, participants assigned to aripiprazole and paliperidone underwent a 1-week tolerability lead-in period with the lowest available oral dose of aripiprazole 2 mg/day or paliperidone 3 mg/day, respectively. Any prior antipsychotic medications were discontinued completely during this period. At Week 1, participants were re-assessed by attending psychiatrists blinded to treatment allocation for self-reported intolerability. Dosages of both medications were then titrated according to clinical response to the psychotic symptoms over the following 3 weeks and subsequently maintained within the recommended therapeutic range for schizophrenia, using either oral or long-acting injectable (LAI) formulations approved by the U.S. Food and Drug Administration (Figure 1). In the TAU group, treatments were determined by the attending psychiatrists according to routine clinical practice as recommended in the United Kingdom National Institute for Health and Care Excellence guidelines (National Institute for Health and Care Excellence, 2011; National Institute for Health and Care Excellence, 2016) and the Royal College of Psychiatrists report (Royal College of Psychiatrists, 2025). Participants could therefore be antipsychotic-free or receive any antipsychotic medication except aripiprazole or paliperidone.

All study medications were supplied and dispensed through three hospital pharmacies in Hong Kong. All medical records, including prescription and dispensing records were digitalized and stored in a centralized electronic clinical database. Oral medication adherence was assessed using pharmacy dispensing records and psychiatrist reports during follow-up visits. LAI formulations were administered on-site by nurses independent of the study team.

### 2.4 Outcome assessments

The primary outcome was the efficacy of aripiprazole and paliperidone compared with TAU in the management of StIP, evaluated using the Clinical Global Impression scales (CGI) (Busner and Targum, 2007): severity (CGI-S), improvement (CGI-I), and efficacy index (CGI-E). Secondary outcomes included severity of StIP psychopathology (Brief Psychiatric Rating Scale [BPRS-24]) (Overall and Gorham, 1962), antipsychotic side-effects (Glasgow Antipsychotic Side-effect Scale [GASS]) (Waddell and Taylor, 2008), severity of stimulant use disorder (Structured Clinical Interview for DSM-5 Disorders–Clinician Version [SCID-5-CV]) (First et al., 2016), psychological dependence (Severity of Dependence Scale [SDS]) (Gossop et al., 1995), treatment motivation (Stages of Change Readiness and Treatment Eagerness Scale–Drugs, [SOCRATES-D]) (Miller and Tonigan, 1996), stimulant use by urine drug testing (panel included cannabis, ketamine, opioids [heroin, morphine, methadone and codeine], sedatives [barbiturates and benzodiazepines], cocaine, methamphetamine, and their metabolites), cognitive functioning (Montreal Cognitive Assessment [MoCA], Nasreddine et al., 2005; Frontal Assessment Battery [FAB]; Dubois et al., 2000), and psychosocial functioning (Addiction Severity Index [ASI]) (McLellan et al., 1980). Detailed descriptions of all the outcome measures are provided in Supplementary Table S2.

All assessments were conducted at four outpatient psychiatric centres in Hong Kong. Board-certified psychiatrists (authors AKKC, FC, and CKL) confirmed the diagnosis of StIP at baseline using DSM-5 criteria, rated CGI scales and BPRS-24, and evaluated the severity of stimulant use disorder. Trained research staff collected demographic information and substance use history and administered the remaining instruments. Outcome measures were assessed at 1-, 3-, 6-, 12- (primary endpoint), and 24 months (secondary endpoint) after randomization (Figure 1). Conversion from StIP to schizophrenia was determined by the attending psychiatrist according to International Statistical Classification of Diseases and Related Health Problems 10th Revision criteria (ICD-10).

### 2.5 Statistical analysis

Sample size was estimated based on a previous three-arm RCT of aripiprazole in amphetamine dependence (Tiihonen et al., 2007), with a target enrolment of 240 participants to detect the referenced medium effect sizes (α = 0.05, β = 0.20) on the primary outcome. All main analyses followed the intention-to-treat principle and included observations from all randomized participants.

Demographic information was summarized as means and standard deviations for continuous variables and as counts with percentages for categorical variables. Between-group differences at baseline were tested using independent t-tests and Pearson’s chi-square tests where appropriate. Treatment effects were estimated using mixed models for repeated measures (MMRM). The primary estimand was the change from baseline at each time point. Models included fixed effects specified for treatment group, time, baseline, treatment-by-time interactions, and baseline-by-time interactions. An unstructured variance-covariance matrix was set at participant level to account for within-subject correlations across repeated visits. Both treatment and time were modelled as categorical variables, with TAU as the reference group. For CGI-I, CGI-E and GASS that lacked pre-randomization baseline value, baseline terms were omitted. Estimated marginal means were calculated for each treatment-by-time combination, and pre-specified contrasts compared each active treatment with TAU at 12 and 24 months. Standardized effect sizes (Cohen’s d) were computed by dividing the group contrasts by the model residual standard deviation. The primary outcome was the change in CGI scores. Other outcomes were exploratory and thus not adjusted for multiplicity. A logistic mixed-effects model with an analogous fixed-effects structure was used for the binary stimulant urine drug test outcome. No missing data imputation was performed, as MMRM yields valid inferences under missing-at-random assumptions.

Exploratory categorical clinical responder analyses on CGI-S, CGI-I and BPRS-24 for the intention-to-treat population at 12 months and 24 months. Clinically meaningful improvement was defined *a priori* as achieving a CGI-S score ≤3, a CGI-I score ≤3, or a ≥50% reduction from baseline in BPRS-24 total score (Leucht et al., 2005; Leucht et al., 2006). Responder proportions were compared between groups using Pearson’s chi-square tests.

GASS total scores were summarized as means and standard deviations, and individual side-effect domains were reported as counts with percentages across follow-up time points. Between-group differences were examined using Kruskal–Wallis tests and Pearson’s chi-square tests with Bonferroni correction applied where appropriate.

Analyses were conducted in R (version 4.4.1) with the mmrm (version 0.3.14), emmeans (version 1.10.4), and lme4 (version 1.1.35.5) packages. Statistical significance was set at *p* < 0.05 (two-sided).

Conversion from StIP to schizophrenia was defined as a new ICD-10 diagnosis of schizophrenia or schizoaffective disorder during follow-up. Participants with a history of these disorders before enrolment were excluded from this analysis. Conversion rates were compared using Pearson’s chi-square test, and time-to-conversion was summarized descriptively.

## 3 Results

**FIGURE 2:**
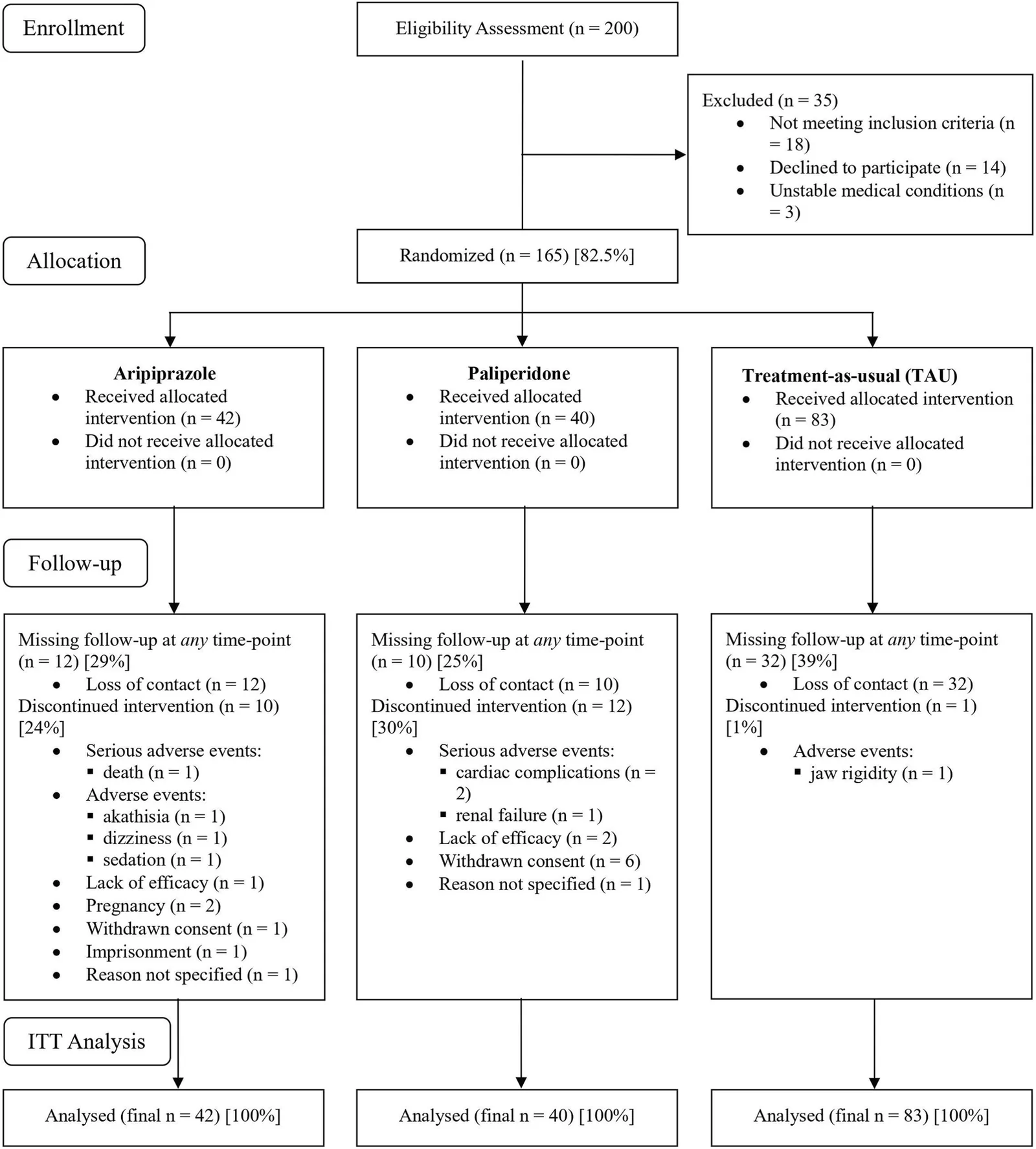
CONSORT diagram.

Recruitment started on June 1, 2019 with the final follow-up completed on May 31, 2024. The COVID-19 pandemic from 2020 to 2023 substantially disrupted recruitment and follow-ups, resulting in a final sample of 165 participants falling short of the target of 240. Figure 2 presents the CONSORT flow diagram, which also summarizes the attrition and adverse events. All 165 randomized participants were included in the intention-to-treat analysis. Assessment completion rates were 70.30% (116/165) at 12 months and 53.94% (89/165) at 24 months, with no significant differences between groups (Supplementary Table S3). No participants received structured psychotherapy or drug counselling during the study.

### 3.1 Baseline characteristics

Participants were predominantly male (69.70%) with a mean age of 38.69 years (SD = 10.08). The aripiprazole group had a lower proportion of men and was younger on average. Other baseline imbalances included higher forensic history but fewer lifetime all-cause psychiatric hospitalizations in the paliperidone group and longer drinking years in the TAU group (Table 1). All participants had used methamphetamine, cocaine or both; 89.70% primarily used methamphetamine, and more than 70% had used it within the past 3 months before enrolment. Mean duration of stimulant use exceeded 6 years. A greater proportion of participants in the aripiprazole group reported lifetime use of both methamphetamine and cocaine than the paliperidone group. At baseline, 96.97% (160/165) provided urine samples, of which 65.63% (105/160) tested positive for stimulants (Supplementary Table S4).

**TABLE 1:** Demographic characteristics, drug use history and study outcomes of 165 participants at baseline.

Baseline characteristics	Total (n = 165)	Aripiprazole (n = 42)	Paliperidone (n = 40)	TAU (n = 83)	p-value^+^
Male (%)	115 (69.70)	19 (45.24)	33 (82.50)	63 (75.90)	PAL > ARI TAU > ARI
Age, mean (SD)	38.69 (10.08)	34.36 (9.98)	39.95 (8.30)	40.28 (10.37)	PAL > ARI
Educational level (%)					
Primary or below	49 (29.70)	12 (28.57)	13 (32.50)	24 (28.92)	​
Secondary	94 (56.97)	25 (59.52)	19 (47.50)	50 (60.24)	​
Tertiary or above	22 (12.58)	5 (11.90)	8 (20.00)	9 (10.84)	​
Marital status (%)					
Single	88 (53.33)	29 (69.05)	19 (47.50)	40 (48.19)	​
Married	32 (19.39)	6 (14.29)	9 (22.50)	17 (20.48)	​
Divorced	45 (27.27)	7 (16.67)	12 (30.00)	26 (31.33)	​
Forensic history (%)	105 (63.64)	20 (47.62)	29 (72.50)	56 (67.47)	PAL > ARI
Tabacco users (%)	147 (90.18)	36 (85.71)	38 (95.00)	73 (90.12)	​
Alcohol users (%)	116 (70.30)	27 (64.29)	26 (65.00)	63 (75.90)	​
Drinking years, mean (SD)	14.54 (12.97)	10.93 (11.64)	15.15 (13.00)	16.07 (13.38)	TAU > ARI
Psychiatric services (%) In-patient Out-patient	117 (70.91) 143 (86.67)	31 (73.81) 36 (85.71)	33 (82.50) 35 (87.50)	53 (63.86) 72 (86.75)	​
Number of all-cause hospitalizations, mean (SD)	5.62 (7.27)	6.18 (6.81)	3.00 (1.63)	6.62 (8.93)	ARI > PAL TAU > PAL
Detox center admission (%)	91 (55.15)	22 (52.38)	21 (51.50)	48 (57.83)	​
Methamphetamine use (%)					
Lifetime use (%)	148 (89.70)	39 (92.86)	36 (90.00)	73 (87.95)	​
Active use in the past 3 months (%)^a^	104 (71.23)	22 (56.41)	29 (82.86)	53 (73.61)	PAL > ARI
Duration of use in months, mean (SD)	125.21 (97.12)	112.39 (87.89)	100.21 (74.28)	144.12 (108.29)	PAL > TAU
Cocaine use (%)					
Lifetime use (%)	98 (59.39)	29 (69.05)	19 (47.50)	50 (60.24)	​
Active use in the past 3 months (%)^a^	42 (42.86)	12 (41.38)	6 (31.58)	24 (48.00)	​
Duration of use in months, mean (SD)	79.67 (85.22)	67.80 (77.19)	72.43 (83.98)	89.08 (90.94)	​
Lifetime use of both stimulants (%)	82 (49.70)	27 (64.29)	15 (37.50)	40 (48.19)	ARI > PAL
Lifetime use of other substances (%)					
Cannabis	123 (74.55)	34 (80.95)	26 (65.00)	63 (75.90)	​
Ketamine	99 (60.00)	29 (59.05)	20 (50.00)	50 (60.24)	​
MDMA	76 (46.06)	22 (52.38)	16 (40.00)	38 (45.78)	​
Zopiclone	66 (40.00)	15 (35.71)	19 (47.50)	32 (38.55)	​
Heroin	46 (27.88)	11 (26.19)	11 (27.50)	24 (28.92)	​
Nimetazepam	35 (21.21)	11 (26.19)	8 (20.00)	16 (19.28)	​
Midazolam	28 (16.97)	6 (14.29)	3 (7.50)	19 (22.89)	​
Methadone	24 (14.55)	4 (9.52)	3 (7.50)	17 (20.48)	​
Outcome measures at baseline					
CGI-S^b^	3.33 (1.29)	3.50 (1.25)	3.45 (1.41)	3.18 (1.25)	​
BPRS-24	29.70 (6.00)	31.55 (6.83)	30.60 (7.21)	28.33 (4.47)	ARI > TAU
Severity of DSM-5 StUD	4.72 (2.27)	5.02 (1.99)	4.41 (2.15)	4.72 (2.45)	​
SDS	5.76 (3.36)	7.24 (3.26)	4.97 (3.08)	5.39 (3.35)	ARI > PAL ARI > TAU
SOCRATES-D	68.81 (12.79)	71.74 (10.29)	70.12 (10.89)	66.70 (14.43)	​
MoCA	24.31 (3.49)	24.24 (4.24)	23.88 (3.62)	24.55 (3.00)	​
FAB	14.92 (4.79)	15.81 (4.75)	14.47 (5.49)	14.69 (4.43)	​
Addiction severity index					
Medical status	0.19 (0.33)	0.24 (0.37)	0.17 (0.31)	0.18 (0.33)	​
Employment/support status	0.78 (0.26)	0.85 (0.22)	0.78 (0.26)	0.75 (0.28)	​
Alcohol	0.08 (0.15)	0.05 (0.10)	0.12 (0.21)	0.08 (0.13)	​
Drugs	0.17 (0.13)	0.15 (0.12)	0.15 (0.12)	0.18 (0.14)	​
Legal status	0.04 (0.12)	0.01 (0.04)	0.07 (0.19)	0.03 (0.09)	​
Family/social relationships	0.18 (0.17)	0.22 (0.20)	0.15 (0.13)	0.18 (0.17)	​
Psychiatric status	0.44 (0.23)	0.53 (0.22)	0.44 (0.23)	0.40 (0.23)	ARI > TAU
Frequency of drug use	6.96 (9.87)	6.71 (9.81)	4.65 (7.80)	8.19 (10.66)	​

ARI: aripiprazole; BPRS-24: Brief Psychiatric Rating Scale–24, items; CGI-S: Clinical Global Impression-Severity; DSM-5, StUD: diagnostic and statistical manual of mental disorders fifth edition stimulant use disorder; FAB: frontal assessment battery; MDMA: 3,4-Methylenedioxymethamphetamine; MoCA: montreal cognitive assessment; n: number of participants; PAL: paliperidone; SD: standard deviation; SDS: severity of dependence scale; SOCRATES: Stages of Change Readiness and Treatment Eagerness Scale–Drugs; TAU: Treatment-as-Usual group.

^a^ Not all participants provided data thus the number of available observations was used as the denominator for percentage calculation.

^b^ CGI-I, and CGI-E, were inherently change scores relative to baseline thus no scorings were made nor reported.

*p-value denotes significant pair-wise comparison between two treatment groups at significance level of 0.05.

### 3.2 Medications and safety

During the Active Intervention phase, 42 participants were assigned to aripiprazole, 40 to paliperidone, and 83 to TAU. No participant reported intolerability to aripiprazole or paliperidone during the 1-week tolerability lead-in phase. Three participants received aripiprazole LAI and ten received paliperidone LAI. Mean oral-equivalent dosages were 15 mg/day (range 2–30) for aripiprazole and 8.1 mg/day (range 3–12) for paliperidone (Supplementary Table S5). During the Observation Maintenance phase, two participants discontinued aripiprazole and remained medication-free, two discontinued paliperidone and switched to quetiapine or olanzapine, and one participant on paliperidone received add-on risperidone (Supplementary Table S6). Antidepressants were the commonest concomitant medications prescribed during the study; however, no significant between group differences were observed in concomitant medication use (Supplementary Table S7). Following randomization, 55.43% of participants in the TAU group were antipsychotic-free at baseline (Supplementary Table S8). This proportion decreased to 31.33% and 24.10% at the end of the Active Intervention phase and the Observation Maintenance phase, respectively.

Four participants (2.42%) experienced serious adverse events (Figure 2). One participant in the aripiprazole group committed suicide 6 months after randomization. Three participants in the paliperidone group discontinued treatment: one due to atrial fibrillation at the 3-month follow-up, one because of corrected QT prolongation (472 m at the 6-month follow-up), and one related to pneumonia-associated acute renal failure (6-month follow-up). No serious adverse event was reported in the TAU group. Other participants in all three treatment groups generally tolerated their assigned interventions well. GASS total scores showed only mild side-effect profiles across groups with no significant between-group differences (Supplementary Table S9). However, paliperidone was associated with higher rates on extrapyramidal side-effects at 24 months (χ2 (2) = 6.73, p = 0.03) and weight gain at 12 months (χ^2^ (2) = 11.00, *p* = 0.004) as compared to TAU. After Bonferroni correction, only the difference in weight gain remained statistically significant (*p* = 0.0009), whereas the extrapyramidal side-effects were no longer significant (*p* = 0.03).

### 3.3 Primary outcome

**FIGURE 3:**
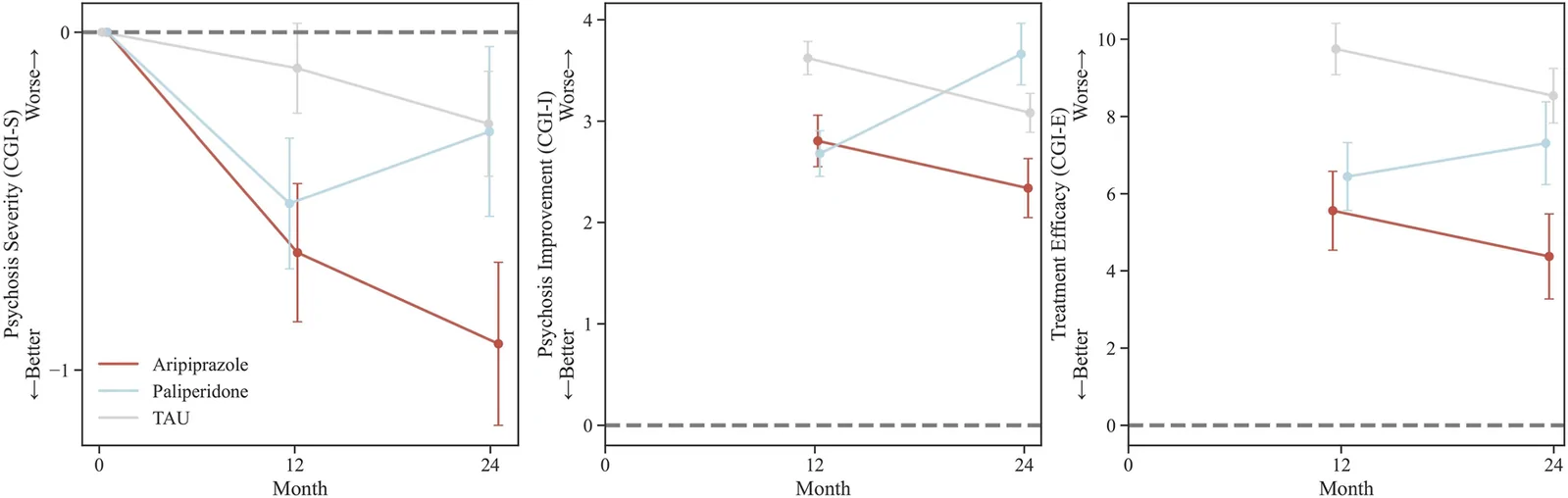
Changes in Clinical Global Impression scores of the three treatment groups on the three subscales at the 12-month primary and the 24-month secondary endpoints.

Table 2 and Figure 3 summarize the changes in CGI scores across the three treatment groups at the 12- and 24-month endpoints. Compared with TAU, only the aripiprazole group demonstrated significant improvement in CGI-S at both 12 months (b = −0.55, 95% CI [−1.03, −0.07], *p* = 0.03, *d* = −0.31) and 24 months (b = −0.65, 95% CI [−1.22, −0.09], p = 0.03, d = −0.38). No significant differences in CGI-S change were observed between the aripiprazole and paliperidone groups. For CGI-I, both the aripiprazole and paliperidone groups improved relative to TAU at 12 months, but only aripiprazole maintained this improvement at 24 months (b = −0.74, 95% CI [−1.43, −0.06], *p* = 0.04, *d* = −0.40). Aripiprazole also showed greater improvement than paliperidone in CGI-I at 24 months (b = −1.32, 95% CI [−2.15, −0.50], *p* < 0.01, *d* = −0.72). A similar pattern was observed for CGI-E, with both groups showing better efficacy than TAU at 12 months, but only aripiprazole maintaining a sustained benefit at 24 months (b = −4.17, 95% CI [−6.73, −1.60], *p* < 0.01, *d* = −1.71). Overall, compared with TAU, aripiprazole was associated with consistent and sustained improvements with small-to-large effect sizes across CGI domains over 24 months.

**TABLE 2:** Changes from baseline in Clinical Global Impression scores for the three subscales across treatment groups at the primary (12-month) and secondary (24-month) endpoints.

Outcome	Endpoint	Contrast	Estimate b [95% CI]	p-value	Cohen’s d
CGI-S	Month 12	Aripiprazole − TAU	−0.55 [-1.03, −0.07]	**0.03**	−0.31
​	​	Paliperidone − TAU	−0.4 [-0.86, 0.06]	0.09	−0.23
​	​	Aripiprazole − paliperidone	−0.15 [-0.7, 0.41]	0.61	−0.08
​	Month 24	Aripiprazole − TAU	−0.65 [-1.22, −0.09]	**0.03**	−0.38
​	​	Paliperidone − TAU	−0.02 [-0.6, 0.56]	0.94	−0.01
​	​	Aripiprazole − paliperidone	−0.63 [-1.31, 0.05]	0.07	−0.36
CGI-I	Month 12	Aripiprazole − TAU	−0.82 [-1.41, −0.23]	**0.01**	−0.45
​	​	Paliperidone − TAU	−0.94 [-1.49, −0.4]	**0.00**	−0.51
​	​	Aripiprazole − paliperidone	0.12 [-0.54, 0.79]	0.71	0.07
​	Month 24	Aripiprazole − TAU	−0.74 [-1.43, −0.06]	**0.04**	−0.40
​	​	Paliperidone − TAU	0.58 [-0.12, 1.28]	0.11	0.32
​	​	Aripiprazole − paliperidone	−1.32 [-2.15, −0.5]	**0.00**	−0.72
CGI-E	Month 12	Aripiprazole − TAU	−4.19 [-6.58, −1.8]	**0.00**	−1.72
​	​	Paliperidone − TAU	−3.3 [-5.47, −1.14]	**0.00**	−1.36
​	​	Aripiprazole − paliperidone	−0.89 [-3.53, 1.76]	0.51	−0.36
​	Month 24	Aripiprazole − TAU	−4.17 [-6.73, −1.6]	**0.00**	−1.71
​	​	Paliperidone − TAU	−1.23 [-3.74, 1.29]	0.34	−0.51
​	​	Aripiprazole − paliperidone	−2.94 [-5.95, 0.07]	0.06	−1.21

CGI-S, Clinical Global Impression-Severity; CGI-I, Clinical Global Impression-Improvement; CGI-E, Clinical Global Impression-Efficacy index; TAU, Treatment-as-usual. Bold value represents statistically significant results with p-value < 0.05.

### 3.4 Secondary outcomes

Paliperidone was associated with a higher proportion of positive urine test for stimulants at 24 months (76.47%) compared with TAU (46.81%; OR = 14.51, 95% CI = [2.07–101.74], *p* < 0.001; Supplementary Table S4). Paliperidone was also associated with a significantly greater decline in global cognitive function, as assessed by MoCA, at 24 months compared with TAU (b = −2.06, 95% CI = [−3.28, −0.85], *p* < 0.001, *d* = −0.97) and aripiprazole (b = −1.57, 95% CI = [−2.97, −0.17], *p* = 0.03, *d* = −0.74) (Supplementary Table S10).

No consistent between-group differences were observed across other secondary outcomes. Severity of StIP psychopathology (BPRS-24), severity of stimulant use disorder (SCID-5-CV), psychological dependence (SDS), frontal executive function (FAB), frequency of stimulant use (ASI), treatment motivation (SOCRATES-D) (Supplementary Table S10; Supplementary Figure S1), and positive urine tests for non-stimulants (Supplementary Table S11) were generally comparable across the three groups. Isolated differences were noted at individual time points with minor psychosocial domains (e.g., legal status), but these effects were not sustained over time.

### 3.5 Clinical responder analysis

Responder rates on CGI-S showed no significant pairwise differences between groups at the 12-month primary or 24-month secondary endpoints (Table 3). In contrast, CGI-I responder rates at 12 months were significantly higher in both aripiprazole and paliperidone groups compared with TAU (both *p* < 0.05), with no significant difference between the two active treatments (*p* = 0.49). These differences were not sustained at 24 months. For BPRS-24, aripiprazole was associated with a significantly higher responder rate than TAU at 12 months (*p* = 0.002), whereas paliperidone did not differ significantly from TAU (*p* = 0.57). No significant between-group differences in BPRS-24 responder rates were observed at 24 months. After Bonferroni correction for multiple comparisons, the differences between aripiprazole and TAU remained statistically significant for both CGI-I (p = 0.0010) and BPRS-24 (*p* = 0.0016) responder rate at 12 months.

**TABLE 3:** Pairwise comparisons of clinical responder rates (%) as defined by CGI-S, CGI-I and BPRS-24 across treatment groups at both primary (12-month) and secondary (24-month) endpoints.

Outcome	Endpoint/Treatment group	Responder rate	Contrast	Chi-square	p-value
CGI-S≤3	Month 12	​	​	​	​
​	Aripiprazole	17/24 (70.83%)	Aripiprazole − TAU	0.36	0.55
​	Paliperidone	21/28 (75.0%)	Paliperidone − TAU	1.06	0.30
​	TAU	41/64 (64.06%)	Aripiprazole − paliperidone	0.11	0.74
​	Month 24	​	​	​	​
​	Aripiprazole	15/21 (71.43%)	Aripiprazole - TAU	0.08	0.76
​	Paliperidone	13/18 (72.22%)	Paliperidone − TAU	0.11	0.74
​	TAU	34/50 (68.0%)	Aripiprazole − paliperidone	0.00	0.96
CGI-I≤3	Month 12	​	​	​	​
​	Aripiprazole	15/24 (71.43%)	Aripiprazole - TAU	10.76	**0.001** ^*^
​	Paliperidone	20/28 (71.43%)	Paliperidone − TAU	17.63	**<0.001** ^*^
​	TAU	16/64 (25%)	Aripiprazole − paliperidone	0.47	0.49
​	Month 24	​	​	​	​
​	Aripiprazole	14/21 (66.67%)	Aripiprazole - TAU	2.53	0.11
​	Paliperidone	7/18 (38.89%)	Paliperidone − TAU	0.27	0.60
​	TAU	23/50 (46.0%)	Aripiprazole − paliperidone	3.01	0.08
BPRS-24 ≥50% reduction	Month 12	​	​	​	​
​	Aripiprazole	16/24 (66.67%)	Aripiprazole - TAU	9.96	**0.002** ^*^
​	Paliperidone	10/28 (35.71%)	Paliperidone − TAU	0.33	0.57
​	TAU	19/64 (29.69%)	Aripiprazole − paliperidone	4.95	**0.03**
​	Month 24	​	​	​	​
​	Aripiprazole	11/21 (52.38%)	Aripiprazole - TAU	0.42	0.52
​	Paliperidone	7/18 (38.89%)	Paliperidone − TAU	0.14	0.71
​	TAU	22/50 (44.02%)	Aripiprazole − paliperidone	0.71	0.40

CGI-S, Clinical Global Impression-Severity; CGI-I, Clinical Global Impression-Improvement; BPRS-24, Brief Psychiatric Rating Scale–24, items; TAU, Treatment-as-usual. Bold value represents statistically significant results with p-value < 0.05. *indicates statistically significant results after Bonferroni correction (adjusted α = 0.0167).

### 3.6 Conversion from StIP to schizophrenia

A *post hoc* retrospective review of electronic health records identified 28 participants (five from aripiprazole, 11 from paliperidone, and 12 from TAU) who had received a diagnosis of schizophrenia or schizoaffective disorders prior to study enrolment. These participants were excluded from the conversion analysis. Among the remaining 137 participants (aripiprazole n = 38, paliperidone n = 29, TAU n = 70), seven converted to schizophrenia during the 24-month follow-up, corresponding to an overall conversion rate of 5.11%. Conversion rates were 7.89% (3/38) in the aripiprazole group, 6.89% (2/29) in the paliperidone group, and 2.86% (2/70) in the TAU group. There was no statistically significant difference between groups (χ^2^ (2) = 1.45, *p* = 0.48). The mean time to conversion was 332 days (SD = 176.83, range 128–611 days).

### 3.7 Sensitivity analysis

Sensitivity analyses restricted to the 137 participants without a prior diagnosis of schizophrenia or schizoaffective disorder demonstrated attenuation effect of the primary outcomes at 12 months (all *p* > 0.05). In contrast, the benefits at 24 months remained statistically significant and were associated with larger effect sizes (p < 0.05, d = −0.39 to −2.13). In the comparison between aripiprazole and TAU, the between-group difference in CGI-S was no longer significant at 12 months (*p* = 0.12) but remained significant at 24 months (*p* = 0.04). For CGI-I, the between-group differences reached only trend-level significance (*p* = 0.07) at 12 months, whereas it remained significant at 24 months (*p* = 0.03). CGI-E findings were unchanged, with significant differences favoring aripiprazole at both 12 and 24 months (both *p* < 0.001). Notably, aripiprazole demonstrated statistically significant superiority over paliperidone on all three CGI subscales showed at 24 months. The significant worsening of global cognitive performance on MoCA in the paliperidone group at 24 months also persisted in this sensitivity analysis (Supplementary Table S12; Supplementary Figure S2).

## 4 Discussion

In this 24-month, two-phase RCT, aripiprazole demonstrated consistently better CGI outcomes compared with TAU in managing StIP. These benefits were observed at the 12-month primary endpoint and 24-month secondary endpoint in the intention-to-treat population, and remained evident at the 24-month secondary endpoint in the sensitivity analysis. This pattern suggests that participants with a pre-existing diagnosis of schizophrenia may have contributed to earlier treatment effects in the primary analysis, while the sustained benefits of aripiprazole at 24 months proved robust to their exclusion. Aripiprazole was also associated with significantly higher responder rates on the CGI-I subscale and BPRS-24 compared with TAU at 12 months. The loss of statistical significance at later time point is likely attributable to reduced statistical power resulting from substantial attrition and smaller sample sizes, although raw responder rates remained numerically highest in the aripiprazole group. In contrast, paliperidone showed only trend-level superiority over TAU at 12 months with no significant benefit at 24 months, and was inferior to aripiprazole at the 24-month endpoint in the sensitivity analysis. Notably, paliperidone was associated with exacerbated stimulant consumption (higher rates of positive urine drug tests) and worsened global cognitive functioning at 24 months. While previous review and meta-analyses of short-term studies have reported broadly comparable efficacy among aripiprazole, haloperidol, olanzapine, paliperidone, quetiapine, and risperidone in treating amphetamine psychosis (Fluyau et al., 2019; Srisurapanont et al., 2021), the present findings concurred with Cuomo et al. that longer-term aripiprazole treatment confers more sustained benefit in StIP (Cuomo et al., 2018).

In the present study, aripiprazole was associated with significant improvements across all three CGI subscales compared with TAU, despite the absence of statistically significant differences in BPRS-24 scores. This divergence reflects fundamental differences in the constructs and scope of the two instruments. The BPRS-24 is a structured, symptom-specific scale that quantifies 24 discrete psychopathological items (positive, negative, and general symptoms) using anchored Likert ratings. While it provides high specificity for core symptomatology, it has limited capacity to integrate broader clinical domains such as functioning, treatment tolerability, or patient wellbeing (Leucht et al., 2005; Leucht and Engel, 2006). In contrast, the CGI subscales (Severity, Improvement, and Efficacy) represent a holistic, clinician-rated global judgment informed by experiential benchmarks within the relevant population. These scales synthesize symptomatic change with overall functioning, side-effect burden, quality of life, and subjective patient status (Leucht et al., 2006). Equipercentile linking studies indicate that CGI ratings correspond more closely to relative (percentage) symptom reductions and are strongly modulated by baseline severity, thereby enabling clinicians to incorporate domains beyond the narrow symptomatic focus of the BPRS-24 (Busner and Targum, 2007; Leucht et al., 2005). This distinction is particularly relevant in our study cohort, which comprised mildly ill participants (mean CGI-S <4) with low baseline BPRS-24 scores (mean < 32). As such, the restricted dynamic range of the BPRS-24 may limit detection of change, whereas even modest improvements in core psychopathology, when accompanied by better tolerability or functional gains, can translate into clinically meaningful differences on CGI assessment. This pattern is further supported by the significantly higher responder rates on both CGI-I and BPRS-24 versus TAU at 12 months. Consequently, the CGI appears more sensitive to clinically relevant differentiations driven by advantages in tolerability or functioning, even when symptomatic efficacy on the BPRS-24 appears comparable. These complementary properties underscore the pragmatic value of the CGI in assessing antipsychotic efficacy while highlighting the need for awareness of its inherent subjectivity.

The contrasting effects of aripiprazole and paliperidone on stimulant use at 24 months may be attributable to their distinct pharmacodynamic profiles. Strong dopamine D2/D3 receptor antagonism and consequent downregulation of D2 receptors in the orbitofrontal cortex and striatum have been implicated in drug craving, enhanced drug rewarding effects and compulsive drug-seeking behavior (Dalley et al., 2007; Lingford-Hughes and Nutt, 2003; Volkow et al., 2015). D2 antagonism in the prefrontal cortex may also exacerbate apathy, motivational deficits and cognitive impairment (Costello et al., 2024; Torrisi et al., 2020). In addition, chronic D2 receptor blockade can induce dopamine super-sensitivity which might potentially aggravating stimulant use (Amarasekera and Wood, 2023). Conversely, 5HT1A receptor agonism is associate with pro-cognitive effects (Sałaciak and Pytka, 2021). Unlike aripiprazole, which possesses both partial D2/D3 and 5HT1A agnostic properties, paliperidone exerts potent D2 antagonism with negligible 5-HT1A activity that may underlie the observed worsening of stimulant use and cognitive functioning (Allott et al., 2023; Citrome et al., 2015; Corena-McLeod, 2015).

The 24-month conversion rate of StIP to schizophrenia in this study was 5.11%, which is substantially lower than the 10%–22% reported in previous observational studies (Murrie et al., 2020; Niemi-Pynttäri et al., 2013). Our findings further suggest that the therapeutic benefits of antipsychotics in StIP may require a longer treatment duration than the commonly recommended 4 weeks to 6 months (Liu, 2021; Wodarz et al., 2017).

### 4.1 Strengths and limitations

Although previous studies have suggested potential benefits of low-dose antipsychotics in StIP, most were short-term and small-scale. The long-term efficacy of antipsychotics in managing StIP, and their impact on concurrent stimulant use and dependence, have remained largely unexamined. To the best of our knowledge, the current study was the first and longest prospective longitudinal RCT evaluating the comparative efficacy and safety of aripiprazole, paliperidone, and TAU in stimulant users with StIP over 2 years. The hybrid study design, comprising a 12-month single-blinded phase followed by a 12-month open-label phase, combined explanatory rigour with pragmatic elements that enhance the generalizability of the findings to real-world clinical practice.

Several limitations should be acknowledged. First, the final sample size was approximately 30% smaller than planned, primarily due to COVID-19-related disruptions. Most participants had moderate stimulant use disorder and mild-to-moderate severity of psychotic symptoms, which may limit extrapolation to more severe presentations of StIP. Second, the heterogeneity of formulations and antipsychotic regimens in the TAU group, together with the uncontrolled concomitant medications, may potentially introduce confounding. Third, the absence of a placebo group precludes evaluation of the natural course of comorbid stimulant use disorder and psychosis, as well as the untreated conversion risk from StIP to schizophrenia. Nevertheless, the use of TAU as an active control in the current study was ethically mandated given the established risks of up to 30% for untreated StIP progressing to schizophrenia (Niemi-Pynttäri et al., 2013). Fourth, the open-label design of the Observation Maintenance phase may have introduced expectation bias in the clinician-rated CGI outcomes, and hence the sustained benefits observed at 24 months should be interpreted with caution. Lastly, the 24-month follow-up duration may be insufficient to fully characterize long-term progression from StIP to schizophrenia. Larger double-blind placebo-controlled trials are needed to determine if early assertive antipsychotic interventions can causally reduce the risk of progression from StIP to schizophrenia. Future studies leveraging large electronic health record databases to identify the most protective antipsychotic treatment strategies are warranted.

### 4.2 Conclusions

Among stimulant users with StIP, aripiprazole demonstrated consistently more favorable clinician-rated outcomes compared with TAU over the 24-month study period. In contrast, paliperidone use may be associated with potential worsening of stimulant use and global cognitive functioning. Side-effects were generally mild across all groups. Large-scale, long-term double-blind, placebo-controlled trials are critically warranted to establish the causal efficacy of early assertive antipsychotic treatments for StIP and in reducing the risk of conversion from StIP to schizophrenia.

## Data Availability

Individual participant data that underlie the results reported in this article after de-identification and the study protocol will be made available beginning 9 months and ending 36 months following the article publication to investigators whose proposed use of the data has been approved by an independent review committee for individual participant data meta-analysis. Proposals should be directed to AKKC and data access would follow the requirements laid in the Policy on the Management of Research Data and Records by the University of Hong Kong. Requests to access the datasets should be directed to chungkka@hku.hk.
