# Supplementary material for: Comparative efficacy of aripiprazole and paliperidone to treatment-as-usual in managing stimulant-induced psychosis: a 24-month randomized controlled trial
Source: Front Pharmacol. 2026 Aug 12;17:1892182. doi: 10.3389/fphar.2026.1892182 (PMC13506957; doi:10.3389/fphar.2026.1892182)
Supplement: Supplementary file 1 [file Supplementaryfile1.docx]

**Supplementary Materials: Comparative efficacy of aripiprazole and paliperidone to treatment-as-usual in managing stimulant-induced psychosis: A 24-month randomized controlled trial**

Supplementary Table 1. Inclusion and exclusion criteria.

Supplementary Table 2. Descriptions of primary and secondary outcome measures.

Supplementary Table 3. Completion rates over the 24-month study period.

Supplementary Table 4. Urine test results for stimulants over the 24-month study period.

Supplementary Table 5. Mean dosages and dosage ranges (mg/day) for prescribed medications during the first 12-month Active Intervention Phase.

Supplementary Table 6. Mean dosages and dosage ranges (mg/day) for prescribed medications during the second 12-month Observation Maintenance Phase (13^th^ to 24^th^ month).

Supplementary Table 7. Number and rate (%) for different classes of medications prescribed in the 3 treatment groups at primary and secondary endpoints.

Supplementary Table 8. Proportions of participants in TAU group remained antipsychotic-free at each follow-up timepoint throughout the study.

Supplementary Table 9. Means and standard deviations (SD) of Glasgow Antipsychotic Side-effect Scale (GASS) scores, and the event counts and rates of different side-effect domains on GASS at all follow-up timepoints in the intention-to-treat sample.

Supplementary Table 10. Pairwise comparisons of secondary outcomes between the three treatment groups at the primary and secondary endpoints.

Supplementary Table 11. Summary of drug urine test results for non-stimulants in the intention-to-treat samples (n=165) at baseline, 12-month primary and 24-month secondary endpoints.

Supplementary Table 12. Pairwise comparisons of all primary and secondary outcomes in the sensitivity analysis (n = 137) with between the three treatment groups at the primary and secondary endpoints.

Supplementary Figure 1. Estimated changes from baseline in primary and secondary outcomes over the two-year study period.

Supplementary Figure 2. Intention-to-treat samples vs sensitivity analysis samples on primary and secondary outcomes over the two-year study period.

**Supplementary Table 1.** Inclusion and exclusion criteria.

| **Inclusion criteria** | **Exclusion criteria** |
| --- | --- |
| - Aged between 16 and 50 years - Able to read and communicate and provide consent in Chinese or English   For participants attending psychiatric services   - diagnosed with stimulant use disorder with co-morbid stimulant-induced psychotic disorders under the Diagnostic and Statistical Manual of Mental Disorders, 5th Edition (DSM-5) - OR stimulant harmful use or dependence syndrome, with co-morbid psychotic disorders or late-onset psychotic disorders due to use of cocaine or other stimulants under the International Classification of Diseases, 10th Revision (ICD-10)   For participants receiving other medical services and/or community agency referrals   - at least two positive urine test results for stimulants within one month - had psychotic symptoms at the time of recruitment | 1. Diagnosed with substance dependence syndrome or use disorder other than stimulants (by DSM-5 or ICD-10) 2. Had been diagnosed with other comorbid psychiatric diagnosis (by DSM-5 or ICD-10)  - intellectual disabilities - other substance-induced mood or psychotic disorders - bipolar disorders, manic episode, or bipolar affective disorders - major depressive disorders with psychotic features - severe depressive episode with psychotic symptoms - recurrent depressive disorders, or current episode severe with psychotic symptoms  3. Taking any maintenance dose of oral antipsychotics continuously ≥12 weeks with psychotic symptoms in remission 4. Receiving any maintenance dose of long-acting injectable depot antipsychotics continuously ≥4 months with psychotic symptoms in remission 5. Other physical conditions  - had suffered from tardive dyskinesia or neuroleptic malignant syndrome before - had known hypersensitivity to risperidone, paliperidone, or aripiprazole - was pregnant or breast-feeding - had a past history of prolonged QTc ≥500ms - known unstable or untreated cardiac disorders - mild to severe renal impairment as indicated by the glomerular filtration rate lower than 80 mL/min |

**Supplementary Table 2.** Descriptions of primary and secondary outcome measures.

| **Primary outcome** | |
| --- | --- |
| 1. Efficacy in stimulant-induced psychosis | Clinician-rated symptom severity, improvement, and overall treatment efficacy were assessed using Clinical Global Impression (CGI) scales. CGI comprises three components: severity (CGI-S), improvement (CGI-I), and efficacy index (CGI-E).  CGI-S evaluates the current severity of participants’ psychotic symptoms on a 7-point Likert scale ranging from 1 (normal, not at all ill) to 7 (among the most extremely ill patients), with higher scores indicating greater severity. A score of 3 or below reflects mild illness, whereas scores of 4 and above indicate increasing levels of severity. A score of 3 or below denotes clinically meaningful improvement treatment responder.  CGI-I assesses change in clinical status relative to baseline on a 7-point scale from 1 (very much improved) to 7 (very much worse), where lower scores indicate greater improvement. A score of 3 or below denotes clinically meaningful improvement (treatment responder).  CGI-E captures the balance between therapeutic effects and side effects on a 16-point scale, ranging from 1 (marked improvement with no side effects) to 16 (no improvement or worsening with side effects outweighing benefits). Although the CGI-E is not strictly linear, it was modeled as a continuous outcome, with lower scores reflecting more favorable treatment efficacy. |
| **Secondary outcomes** | |
| 2. Severity of the stimulant-induced psychosis psychopathology | Brief Psychiatric Rating Scale (BPRS-24) was used to assess participants’ stimulant-induced psychosis psychopathology across 24 distinct constructs, ranging from somatic concern, anxiety, depression, suicidality to motor hyperactivity, and mannerism. This 7-point Likert scale ranges from 1 (not present) and 2 (very mild) to 7 (extremely severe), with greater values indicating more severe psychotic symptomatology overall. A 50% or greater reduction in score from its baseline is considered a clinically significant improvement. |
| 3. Antipsychotic tolerability | All participants self-reported the frequency and severity of experienced side effects of antipsychotic medications using 20-item Glasgow Antipsychotic Side-effect Scale (GASS). The scale probed a wide range of symptoms such as extra pyramidal, gastrointestinal, and cardiovascular side effects. In our analysis, we solely focused on the frequency measures in which each side effect was self-reported by participants on a 4-point Likert scale from 0 (never), 1 (once), 2 (a few times), 3 (everyday), with greater value representing more frequently reported experiences of side effects. A total GASS score of 0-21 is classified as absent or mild side effects, 22-42 indicates moderate side effects, and 43-63 represents severe side effects. |
| 4. Severity of DSM-5 stimulant use disorder | Structured Clinical Interview for DSM-5 Disorders–Clinician Version (SCID-5-CV) was used to systematically assess each participant’s stimulant use symptoms based on the DSM-5 criteria of stimulant use disorder. The assessment identifies 11 possible symptoms of substance-related impairment such as craving, increased consumption, and social and interpersonal problems. Higher numbers of symptoms represent greater severity of stimulant use disorder, which is classified as mild with 2-3 symptoms present, moderate with 4-5 symptoms, and severe with 6 or more symptoms. |
| 5. Psychological dependence to stimulants | The Severity of Dependence Scale (SDS) is a five-item self-report scale assessing psychological dependence on substance, including sense of control, anxiety, worry, desire, and difficulty associated with stop using. Participants answered each item on a 4-point Likert scale from 0 (never) to 3 (always) and the total score was 15 with higher numbers signaling greater stimulant dependence. A total SDS score above 3 marks the clinical threshold of substance dependence. |
| 6. Motivation to change for stimulant use | The Stages of Change Readiness and Treatment Eagerness Scale–Drug (SOCRATES-D) is a 19-item self-report scale assessing participants’ readiness to change their drug use behaviors. The scale consists of three subcomponents: recognition, ambivalence, and taking steps. Participants answered each item on a 5-point Likert scale from 1 (strongly disagree) to 5 (strongly agree). A sum score of each component was taken (range 19-95) with higher scores indicating greater level of willingness to change. |
| 7. Urine drug testing | At each visit, participants did a urine test (by either a urine test kit or laboratory urine test) for detecting the presence of cannabis, ketamine, opioids (heroin, morphine, methadone and codeine), sedatives (barbiturates and benzodiazepines), methamphetamine/ cocaine, and their metabolites. The result of each urine test was coded dichotomously (Positive = 1, Negative = 0) if any one of the tested substance or metabolites was found to be positive. |
| 8. Cognitive functioning | Trained research staff administered Montreal Cognitive Assessment (MoCA) and Frontal Assessment Battery (FAB) to monitor participant’s cognitive performance. MoCA assesses functions ranging from memory, language, visuospatial skills, executive function, with a total score of 30. A MoCA score below 26 suggests mild cognitive impairment, and 24 or lower indicates the presence of neurocognitive disorders. FAB assesses frontal executive functions with six sub-tests: conceptualization, mental flexibility, motor programming, sensitivity to interference, inhibitory control, and environmental autonomy, with a total score of 18. A FAB score of 12 or below suggests a dysexecutive cognitive phenotype and indicates impairment in frontal executive functioning. |
| 9. Psychosocial functioning | Trained research staff conducted a semi-structured interview evaluating multiple areas affected by substance use with Addiction Severity Index (ASI-Lite). The domains of social and functional measures include medical, employment, alcohol, drug, legal, family/social, psychiatric, and frequency of drug use in the past 30 days. Each component yields a composite score between 0 and 1, with higher scores indicating greater impairment except for employment. |

**Supplementary Table 3.** Completion rates over the 24-month study period.

|  | Total | Aripiprazole | Paliperidone | TAU | *χ*² | p-value |
| --- | --- | --- | --- | --- | --- | --- |
| Baseline | 165 (100%) | 42 (100%) | 40 (100%) | 83 (100%) |  | 1.00 |
| Month 1 | 111 (67.27%) | 30 (71.43%) | 29 (72.50%) | 52 (62.65%) | 1.63 | 0.44 |
| Month 3 | 119 (72.12%) | 32 (76.19%) | 31 (77.50%) | 56 (67.47%) | 1.82 | 0.40 |
| Month 6 | 113 (68.48%) | 29 (69.05%) | 28 (70.00%) | 56 (67.47%) | 0.09 | 0.96 |
| Month 12 | 116 (70.30%) | 24 (57.14%) | 28 (70.00%) | 64 (77.11%) | 5.33 | 0.07 |
| Month 24 | 89 (53.94%) | 21 (50.00%) | 18 (45.00%) | 50 (60.24%) | 2.88 | 0.24 |

Note. TAU: treatment-as-usual.

**Supplementary Table 4.** Urine test results for stimulants over the 24-month study period.

|  | Total | Aripiprazole | Paliperidone | TAU |
| --- | --- | --- | --- | --- |
| Baseline | 105/160 (65.62%) | 24/39 (58.33%) | 22/38 (56.76%) | 59/83 (70.27%) |
| Month 12 | 66/113 (58.41%) | 12/23 (52.17%) | 18/27 (66.67%) | 36/63 (57.14%) |
| Month 24 | 45/83 (54.22%) | 10/19 (52.63%) | 13/17 (76.47%) | 22/47 (46.81%) |

Note. Numerator represents the number of positive urine test results for stimulants; denominator represents the total number of urine sample collected. TAU: treatment-as-usual.

**Supplementary Table 5**. Mean dosages and dosage ranges (mg/day) for prescribed medications during the first 12-month Active Intervention Phase.

|  | Aripiprazole | Paliperidone | TAU |
| --- | --- | --- | --- |
| Antipsychotics |  |  |  |
| Aripiprazole (oral/LAI*) | 15 (2–30) | .. | .. |
| Paliperidone (oral/LAI*) | .. | 8.1 (3–12) | .. |
| Amisulpride | .. | .. | 900 (900–900) |
| Brexpiprazole | .. | .. | 1 (1–1) |
| Haloperidol LAI* | .. | .. | 3.33 (3.33–3.33) |
| Lurasidone | .. | .. | 160 (160–160) |
| Olanzapine | .. | .. | 9.7 (5–20) |
| Quetiapine | 39.1 (12.5–100) | 104.5 (25–100) | 191.1 (25–750) |
| Risperidone | .. | .. | 1.3 (1–2) |
| Sulpiride | .. | .. | 400 (400–400) |
| Ziprasidone | .. | .. | 80 (40–120) |
| Trifluoperazine | .. | .. | 3 (3–3) |
| Clopixol LAI* | .. | .. | 55 (40–70) |
| Antidepressant | | | |
| Desvenlafaxine | 62.5 (25–100) | 66.7 (50–100) | 66.7 (50–100) |
| Escitalopram | .. | .. | 20 (20–20) |
| Mirtazapine | 37.5 (37.5–37.5) | .. | 15 (15–15) |
| Venlafaxine | 37.5 (37.5–37.5) | .. | .. |
| Sertraline | 100 (100–100) | 58.3 (50–75) | 70.8 (25–150) |
| Trazodone | 112.5 (50–200) | 87.5 (75–100) | 165 (25–300) |
| Mood-stabilizing agents | | | |
| Lithium | .. | 400 (400–400) | .. |
| Lamotrigine | 25 (25–25) | .. | .. |
| Sodium valproate | 1000 (1000–1000) | 500 (500–500) | 600 (500–700) |
| Adjunctive medication |  |  |  |
| Trihexyphenidyl | 4.2 (2–12) | 5.1 (2–12) | 4.4 (2–6) |
| Propranolol | 30 (30–30) | 20 (20–20) | 14 (10–20) |
| Diazepam | 9 (9–9) | 5.7 (5–7) | 6 (2–10) |

Note. * LAI dosages were converted to oral equivalent doses according to the prescription information and literature. LAI: long-acting injectable; TAU: treatment-as-usual.

**Supplementary Table 6**. Mean dosages and dosage ranges (mg/day) for prescribed medications during the second 12-month Observation Maintenance Phase (13^th^ to 24^th^ month).

|  | Aripiprazole | Paliperidone | TAU |
| --- | --- | --- | --- |
| Antipsychotics |  |  |  |
| Aripiprazole (oral/LAI*) | 15.5 (5–30) | .. | 2.5 (2-3) |
| Paliperidone (oral/LAI*) | .. | 8.4 (3–12) | .. |
| Brexpiprazole | .. | .. | 3 (1–4) |
| Lurasidone | .. | .. | 120 (120–120) |
| Olanzapine | .. | 20 (20-20) | 12.1 (5–20) |
| Quetiapine | 51.8 (12.5–100) | 106.8 (25–250) | 238.9 (25–750) |
| Risperidone |  | 1 (1–1) | .. |
| Sulpiride | .. | .. | 400 (400–400) |
| Ziprasidone | .. | .. | 75 (40–120) |
| Trifluoperazine | .. | .. | 2 (2–2) |
| Clopixol LAI* | .. | .. | 55 (30–80) |
| Antidepressant | | | |
| Citalopram | .. | .. | 20 (20–20) |
| Desvenlafaxine | 75 (50–100) | 75 (75–75) | 75 (50–100) |
| Escitalopram | .. | .. | 20 (20–20) |
| Mirtazapine | 37.5 (37.5–37.5) | .. | 30 (15–45) |
| Sertraline | 100 (100–100) | 75 (75–75) | 75 (25–150) |
| Trazodone | 112.5 (75–200) | .. | 162.5 (100–250) |
| Mood-stabilizing agents | | | |
| Lamotrigine | 150 (150–150) | .. | .. |
| Sodium valproate | 1000 (1000–1000) | .. | 750 (500–1000) |
| Adjunctive medication |  |  |  |
| Trihexyphenidyl | 4.7 (2–12) | 3.5 (2–4) | 3.9 (2–8) |
| Propranolol | 26.7 (20–30) | 15 (10–20) | 13.3 (10–20) |
| Diazepam | 9 (9–9) | .. | 3 (2–5) |

Note. * LAI dosages were converted to oral equivalent doses according to the prescription information and literature. LAI: long-acting injectable; TAU: treatment-as-usual.

**Supplementary Table 7.** Number and rate (%) for different classes of medications prescribed in the 3 treatment groups at primary and secondary endpoints.

| Month | Medication Class | Group | Number of participants completed follow up | Count (%) | Chi-square | p-value |
| --- | --- | --- | --- | --- | --- | --- |
| 12 |  |  | 116 |  |  |  |
|  | Antidepressant |  |  | 30 (25.86%) | 0.17 | 0.92 |
|  |  | Aripiprazole | 24 | 7 (29.2%) |  |  |
|  |  | Paliperidone | 28 | 7 (25.0%) |  |  |
|  |  | TAU | 64 | 16 (25.0%) |  |  |
|  | Mood stabilizer |  |  | 6 (5.17%) | 1.26 | 0.53 |
|  |  | Aripiprazole | 24 | 2 (8.3%) |  |  |
|  |  | Paliperidone | 28 | 2 (7.1%) |  |  |
|  |  | TAU | 64 | 2 (3.1%) |  |  |
|  | Benzodiazepine |  |  | 6 (5.17%) | 2.35 | 0.31 |
|  |  | Aripiprazole | 24 | 1 (4.2%) |  |  |
|  |  | Paliperidone | 28 | 3 (10.7%) |  |  |
|  |  | TAU | 64 | 2 (3.1%) |  |  |
|  | Trihexyphenidyl |  |  | 26 (22.41%) | 4.94 | 0.08 |
|  |  | Aripiprazole | 24 | 9 (37.5%) |  |  |
|  |  | Paliperidone | 28 | 7 (25.0%) |  |  |
|  |  | TAU | 64 | 10 (15.6%) |  |  |
|  | Propranolol |  |  | 7 (6.03%) | 0.80 | 0.67 |
|  |  | Aripiprazole | 24 | 1 (4.2%) |  |  |
|  |  | Paliperidone | 28 | 1 (3.6%) |  |  |
|  |  | TAU | 64 | 5 (7.8%) |  |  |
| 24 |  |  | 89 |  |  |  |
|  | Antidepressant |  |  | 25 (28.09%) | 3.23 | 0.20 |
|  |  | Aripiprazole | 21 | 7 (33.3%) |  |  |
|  |  | Paliperidone | 18 | 2 (11.1%) |  |  |
|  |  | TAU | 50 | 16 (32.0%) |  |  |
|  | Mood Stabilizer |  |  | 4 (4.49%) | 2.24 | 0.33 |
|  |  | Aripiprazole | 21 | 2 (9.5%) |  |  |
|  |  | Paliperidone | 18 | 0 (0.0%) |  |  |
|  |  | TAU | 50 | 2 (4.0%) |  |  |
|  | Benzodiazepine |  |  | 4 (4.49%) | 1.11 | 0.57 |
|  |  | Aripiprazole | 21 | 1 (4.8%) |  |  |
|  |  | Paliperidone | 18 | 0 (0.0%) |  |  |
|  |  | TAU | 50 | 3 (6.0%) |  |  |
|  | Trihexyphenidyl |  |  | 19 (21.35%) | 0.99 | 0.61 |
|  |  | Aripiprazole | 21 | 6 (28.6%) |  |  |
|  |  | Paliperidone | 18 | 4 (22.2%) |  |  |
|  |  | TAU | 50 | 9 (18.0%) |  |  |
|  | Propranolol |  |  | 8 (8.99%) | 1.37 | 0.51 |
|  |  | Aripiprazole | 21 | 3 (14.3%) |  |  |
|  |  | Paliperidone | 18 | 2 (11.1%) |  |  |
|  |  | TAU | 50 | 3 (6.0%) |  |  |

**Supplementary Table 8.** Proportions of participants in TAU group (n = 83) remained antipsychotic-free at each follow-up timepoint throughout the study.

| Group | Month | Number of participants remained antipsychotics-free |
| --- | --- | --- |
| TAU | 0 | 46 (55.4%) |
| TAU | 1 | 20 (24.10%) |
| TAU | 3 | 25 (30.12%) |
| TAU | 6 | 31 (37.35%) |
| TAU | 12 | 26 (31.33%) |
| TAU | 24 | 20 (24.10%) |

**Supplementary Table 9.** Means and standard deviations (SD) of Glasgow Antipsychotic Side-effect Scale (GASS) scores, and the event counts and rates of different side-effect domains on GASS at all follow-up timepoints in the intention-to-treat sample (n = 165).

|  | Treatment group | Scores at follow-up timepoints  (Mean [SD]) | | | | | Count and rate (%) of any symptom reported | | | | |
| --- | --- | --- | --- | --- | --- | --- | --- | --- | --- | --- | --- |
|  |  | Month 1 | Month 3 | Month 6 | Month 12 | Month 24 | Month 1 | Month 3 | Month 6 | Month 12 | Month 24 |
| GASS total score | Aripiprazole | 15.3 (9.5) | 14.8 (8.6) | 14.4 (10.4) | 10.5 (7.0) | 13.7 (9.2) | 29/30 (97%) | 30/32 (94%) | 27/29 (93%) | 24/24 (100%) | 21/21 (100%) |
|  | Paliperidone | 13.1 (10.0) | 12.3 (11.0) | 14.2 (11.3) | 12.7 (9.5) | 12.1 (8.0) | 28/29 (97%) | 29/31 (94%) | 26/28 (93%) | 27/28 (96%) | 17/18 (94%) |
|  | TAU | 11.4 (9.2) | 11.7 (9.4) | 12.7 (9.5) | 12.0 (9.9) | 11.1 (10.1) | 47/52 (90%) | 48/56 (86%) | 53/56 (95%) | 59/64 (92%) | 44/50 (88%) |
|  |  | H=4.40,  *p* = 0.11 | H=3.43,  *p* = 0.18 | H=0.63,  *p* =0.73 | H=0.46,  *p* =0.80 | H=2.06,  *p* = 0.36 | χ^2^ = 1.81;  *p* = 0.40 | χ^2^ = 2.06;  *p* = 0.36 | χ^2^ = 0.14;  *p* = 0.94 | χ^2^ = 2.36;  *p* = 0.31 | χ^2^ = 3.10;  *p* = 0.21 |
| Domains | | | | | | | | | | | |
| Sedation and CNS side effects  (Q1-2) | Aripiprazole | 3.0 (2.2) | 2.4 (2.0) | 2.7 (2.1) | 2.2 (1.8) | 2.5 (1.9) | 24/30 (80%) | 22/32 (69%) | 22/29 (76%) | 18/24 (75%) | 16/21 (76%) |
|  | Paliperidone | 2.4 (2.1) | 2.1 (2.1) | 2.6 (2.3) | 2.3 (1.8) | 2.1 (1.8) | 20/29 (69%) | 19/31 (61%) | 19/28 (68%) | 20/28 (71%) | 14/18 (78%) |
|  | TAU | 2.0 (1.9) | 2.1 (1.9) | 2.4 (1.8) | 2.4 (1.9) | 1.7 (1.9) | 34/52 (65%) | 35/56 (62%) | 44/56 (79%) | 48/64 (75%) | 29/50 (58%) |
|  |  |  |  |  |  |  | χ^2^ = 1.98;  *p* = 0.37 | χ^2^ = 0.47;  *p* = 0.79 | χ^2^ = 1.16;  *p* = 0.56 | χ^2^ = 0.14;  *p* = 0.93 | χ^2^ = 3.52;  *p* = 0.17 |
|  | | | | | | | | | | | |
| Cardiovascular side-effects  (Q3-4) | Aripiprazole | 1.7 (1.9) | 1.6 (1.8) | 1.6 (1.5) | 0.9 (1.2) | 1.7 (1.7) | 18/30 (60%) | 19/32 (59%) | 18/29 (62%) | 10/24 (42%) | 14/21 (67%) |
|  | Paliperidone | 1.3 (1.8) | 1.1 (1.3) | 1.3 (1.7) | 1.2 (1.5) | 1.6 (1.5) | 14/29 (48%) | 15/31 (48%) | 15/28 (54%) | 14/28 (50%) | 12/18 (67%) |
|  | TAU | 1.2 (1.4) | 1.1 (1.6) | 1.0 (1.3) | 1.1 (1.5) | 0.9 (1.1) | 29/52 (56%) | 24/56 (43%) | 25/56 (45%) | 28/64 (44%) | 24/50 (48%) |
|  |  |  |  |  |  |  | χ^2^ = 0.85;  *p* = 0.66 | χ^2^ = 2.23;  *p* = 0.33 | χ^2^ = 2.40;  *p* = 0.30 | χ^2^ = 0.43;  *p* = 0.81 | χ^2^ = 3.10;  *p* = 0.21 |
|  | | | | | | | | | | | |
| Extra-pyramidal side-effects  (Q5-10) | Aripiprazole | 5 (4.1) | 4.9 (3.5) | 4.6 (4.7) | 2.8 (2.9) | 3.7 (3.9) | 27/30 (90%) | 27/32 (84%) | 21/29 (72%) | 16/24 (67%) | 15/21 (71%) |
|  | Paliperidone | 4.0 (3.8) | 3.8 (4.3) | 4.3 (4.5) | 3.7 (4.0) | 3.5 (2.3) | 21/29 (72%) | 22/31 (71%) | 21/28 (75%) | 21/28 (75%) | 16/18 (89%) |
|  | TAU | 2.9 (3.1) | 3.1 (3.2) | 3.5 (3.6) | 3.6 (3.5) | 3.0 (3.8) | 37/52 (71%) | 39/56 (70%) | 43/56 (77%) | 44/64 (69%) | 28/50 (56%) |
|  |  |  |  |  |  |  | χ^2^ = 4.15;  *p* = 0.13 | χ^2^ = 2.49;  *p* = 0.29 | χ^2^ = 0.20;  *p* = 0.91 | χ^2^ = 0.50;  *p* = 0.78 | **χ^2^ = 6.73;**  ***p* = 0.03** |
|  | | | | | | | | | | | |
| Anticholinergic side-effects  (Q11-13) | Aripiprazole | 2.6 (2.3) | 2.6 (2.5) | 2.6 (2.3) | 2 (1.9) | 2.3 (2.1) | 22/30 (73%) | 22/32 (69%) | 21/29 (72%) | 16/24 (67%) | 14/21 (67%) |
|  | Paliperidone | 2.2 (2.2) | 2.2 (2.4) | 2.6 (2.2) | 2.3 (2.6) | 1.9 (1.9) | 20/29 (69%) | 21/31 (68%) | 20/28 (71%) | 17/28 (61%) | 12/18 (67%) |
|  | TAU | 1.8 (1.7) | 2.1 (2.2) | 2.4 (2.3) | 2.2 (2.4) | 2 (2.2) | 33/52 (63%) | 36/56 (64%) | 39/56 (70%) | 42/64 (66%) | 31/50 (62%) |
|  |  |  |  |  |  |  | χ^2^ = 0.88;  *p* = 0.64 | χ^2^ = 0.25;  *p* = 0.88 | χ^2^ = 0.08;  *p* = 0.96 | χ^2^ = 0.26;  *p* = 0.86 | χ^2^ = 0.21;  *p* = 0.90 |
|  | | | | | | | | | | | |
| Gastro-intestinal side-effects  (Q14) | Aripiprazole | 0.4 (0.7) | 0.3 (0.7) | 0.5 (0.7) | 0.2 (0.6) | 0.3 (0.6) | 8/30 (27%) | 8/32 (25%) | 10/29 (34%) | 3/24 (13%) | 4/21  (19%) |
|  | Paliperidone | 0.2 (0.6) | 0.2 (0.6) | 0.4 (0.9) | 0.3 (0.6) | 0.3 (0.6) | 5/29 (17%) | 5/31 (16%) | 5/28  (18%) | 7/28 (25%) | 4/18  (22%) |
|  | TAU | 0.3 (0.6) | 0.2 (0.6) | 0.5 (0.8) | 0.2 (0.6) | 0.3 (0.7) | 11/52 (21%) | 9/56 (16%) | 16/56 (29%) | 8/64 (13%) | 10/50 (20%) |
|  |  |  |  |  |  |  | χ^2^ = 0.79;  *p* = 0.68 | χ^2^ = 1.25;  *p* = 0.54 | χ^2^ = 2.05;  *p* = 0.36 | χ^2^ = 2.53;  *p* = 0.28 | χ^2^ = 0.06;  *p* = 0.97 |
|  | | | | | | | | | | | |
| Genitourinary side-effects  (Q15) | Aripiprazole | 0 (0) | 0 (0) | 0.0 (0.2) | 0 (0) | 0 (0) | 0/30 (0%) | 0/32 (0%) | 1/29 (3%) | 0/24 (0%) | 0/21 (0%) |
|  | Paliperidone | 0.2 (0.5) | 0.1 (0.6) | 0.1 (0.5) | 0.0 (0.2) | 0.2 (0.5) | 3/29 (10%) | 2/31 (6%) | 2/28 (7%) | 1/28 (4%) | 2/18 (11%) |
|  | TAU | 0.1 (0.3) | 0.1 (0.3) | 0.2 (0.5) | 0.0 (0.3) | 0.1 (0.3) | 2/52 (4%) | 2/56 (4%) | 6/56 (11%) | 2/64 (3%) | 4/50 (8%) |
|  |  |  |  |  |  |  | χ^2^ = 3.77;  *p* = 0.15 | χ^2^ = 2.03;  *p* = 0.36 | χ^2^ = 1.41;  *p* = 0.49 | χ^2^ = 0.88;  *p* = 0.64 | χ^2^ = 2.19;  *p* = 0.33 |
|  | | | | | | | | | | | |
| Diabetes mellitus  (Q16) | Aripiprazole | 0.7 (1.0) | 0.8 (1.1) | 0.8 (1.1) | 0.8 (1.1) | 0.9 (1.1) | 12/30 (40%) | 14/32 (44%) | 12/29 (41%) | 10/24 (42%) | 9/21 (43%) |
|  | Paliperidone | 0.7 (1.2) | 0.9 (1.2) | 1.0 (1.2) | 0.7 (1.0) | 0.6 (1.1) | 9/29 (31%) | 14/31 (45%) | 13/28 (46%) | 12/28 (43%) | 5/18 (28%) |
|  | TAU | 1.0 (1.1) | 1.0 (1.1) | 0.8 (1.0) | 0.8 (1.0) | 0.7 (1.0) | 27/52 (52%) | 28/56 (50%) | 24/56 (43%) | 30/64 (47%) | 20/50 (40%) |
|  |  |  |  |  |  |  | χ^2^ = 3.49;  *p* = 0.18 | χ^2^ = 0.38;  *p* = 0.83 | χ^2^ = 0.19;  *p* = 0.91 | χ^2^ = 0.25;  *p* = 0.88 | χ^2^ = 1.09;  *p* = 0.58 |
|  | | | | | | | | | | | |
| Prolactinaemic side-effects  (Q17-21) | Aripiprazole | 1 (1.9) | 1.1 (1.7) | 0.7 (1.5) | 0.7 (1.3) | 1.0 (1.6) | 9/30 (30%) | 12/32 (38%) | 7/29 (24%) | 6/24 (25%) | 8/21 (38%) |
|  | Paliperidone | 0.6 (1.3) | 0.8 (1.7) | 0.7 (1.8) | 0.5 (1.0) | 1.1 (1.7) | 7/29 (24%) | 8/31 (26%) | 6/28 (21%) | 6/28 (21%) | 7/18 (39%) |
|  | TAU | 0.8 (1.7) | 1.0 (1.7) | 1.2 (1.9) | 1.0 (1.7) | 1.3 (2.0) | 13/52 (25%) | 21/56 (38%) | 22/56 (39%) | 21/64 (33%) | 21/50 (42%) |
|  |  |  |  |  |  |  | χ^2^ = 0.36;  *p* = 0.84 | χ^2^ = 1.39;  *p* = 0.50 | χ^2^ = 3.58;  *p* = 0.17 | χ^2^ = 1.42;  *p* = 0.49 | χ^2^ = 0.12;  *p* = 0.94 |
|  | | | | | | | | | | | |
| Weight gain  (Q22) | Aripiprazole | 0.9 (1.4) | 1.0 (1.4) | 0.9 (1.4) | 1.0 (1.4) | 1.3 (1.5) | 9/30 (30%) | 11/32 (34%) | 9/29 (31%) | 8/24 (33%) | 9/21 (43%) |
|  | Paliperidone | 1.3 (1.5) | 1.0 (1.4) | 1.2 (1.5) | 1.7 (1.5) | 0.8 (1.4) | 13/29 (45%) | 10/31 (32%) | 12/28 (43%) | 16/28 (57%) | 5/18 (28%) |
|  | TAU | 1.3 (1.5) | 1.1 (1.5) | 0.9 (1.4) | 0.7 (1.3) | 1.0 (1.4) | 23/52 (44%) | 20/56 (36%) | 17/56 (30%) | 14/64 (22%) | 16/50 (32%) |
|  |  |  |  |  |  |  | χ^2^ = 1.91;  *p* = 0.39 | χ^2^ = 0.10;  *p* = 0.95 | χ^2^ = 1.42;  *p* = 0.49 | **χ^2^ = 11.00;**  ***p* = 0.004** | χ^2^ = 1.14;  *p* = 0.57 |

Note: Counts were compared using chi-square test (df = 2) and means using Kruskal–Wallis tests. CNS: central nervous system; Q: question in GASS; TAU: treatment-as-usual.

**Supplementary Table 10.** Pairwise comparisons of secondary outcomes in the ITT analysis (n = 165) between the three treatment groups at the primary and secondary endpoints.

|  | Endpoint | Contrast | Estimate b [95%CI] | p-value | Cohen’s d |
| --- | --- | --- | --- | --- | --- |
| BPRS-24 | Month 12 | Aripiprazole - TAU | -0.89 [-3.47, 1.69] | 0.5 | -0.36 |
|  | Month 12 | Paliperidone - TAU | -1.08 [-3.53, 1.37] | 0.39 | -0.44 |
|  | Month 12 | Aripiprazole - Paliperidone | 0.19 [-2.71, 3.09] | 0.9 | 0.08 |
|  | Month 24 | Aripiprazole - TAU | -0.6 [-3.29, 2.09] | 0.66 | -0.24 |
|  | Month 24 | Paliperidone - TAU | 1.03 [-1.78, 3.84] | 0.47 | 0.42 |
|  | Month 24 | Aripiprazole - Paliperidone | -1.63 [-4.88, 1.62] | 0.33 | -0.66 |
|  |  |  |  |  |  |
| GASS | Month 12 | Aripiprazole - TAU | -1.4 [-5.29, 2.49] | 0.48 | -0.53 |
|  | Month 12 | Paliperidone - TAU | 0.05 [-3.74, 3.85] | 0.98 | 0.02 |
|  | Month 12 | Aripiprazole - Paliperidone | -1.45 [-5.94, 3.03] | 0.53 | -0.55 |
|  | Month 24 | Aripiprazole - TAU | 3.87 [-0.39, 8.14] | 0.08 | 1.46 |
|  | Month 24 | Paliperidone - TAU | 0.83 [-3.66, 5.31] | 0.72 | 0.31 |
|  | Month 24 | Aripiprazole - Paliperidone | 3.05 [-2.13, 8.22] | 0.25 | 1.15 |
|  |  |  |  |  |  |
| Severity of DSM-5 StUD | Month 12 | Aripiprazole - TAU | 0.31 [-0.86, 1.48] | 0.6 | 0.15 |
|  | Month 12 | Paliperidone - TAU | 0.27 [-0.87, 1.41] | 0.65 | 0.13 |
|  | Month 12 | Aripiprazole - Paliperidone | 0.05 [-1.33, 1.42] | 0.95 | 0.02 |
|  | Month 24 | Aripiprazole - TAU | -0.43 [-1.95, 1.08] | 0.58 | -0.2 |
|  | Month 24 | Paliperidone - TAU | 0.24 [-1.45, 1.93] | 0.78 | 0.11 |
|  | Month 24 | Aripiprazole - Paliperidone | -0.67 [-2.62, 1.28] | 0.5 | -0.32 |
|  |  |  |  |  |  |
| SDS | Month 12 | Aripiprazole - TAU | 0.05 [-1.19, 1.3] | 0.94 | 0.02 |
|  | Month 12 | Paliperidone - TAU | 0.56 [-0.59, 1.71] | 0.34 | 0.25 |
|  | Month 12 | Aripiprazole - Paliperidone | -0.51 [-1.94, 0.92] | 0.49 | -0.23 |
|  | Month 24 | Aripiprazole - TAU | 1.44 [-0.15, 3.03] | 0.08 | 0.65 |
|  | Month 24 | Paliperidone - TAU | 1.55 [-0.06, 3.17] | 0.06 | 0.7 |
|  | Month 24 | Aripiprazole - Paliperidone | -0.12 [-2.02, 1.78] | 0.9 | -0.05 |
|  |  |  |  |  |  |
| SOCRATES-D | Month 12 | Aripiprazole - TAU | 1.18 [-3.1, 5.45] | 0.59 | 0.43 |
|  | Month 12 | Paliperidone - TAU | -2.4 [-6.44, 1.64] | 0.25 | -0.87 |
|  | Month 12 | Aripiprazole - Paliperidone | 3.58 [-1.2, 8.36] | 0.14 | 1.29 |
|  | Month 24 | Aripiprazole - TAU | -0.9 [-8.12, 6.32] | 0.81 | -0.32 |
|  | Month 24 | Paliperidone - TAU | -4.64 [-12.17, 2.89] | 0.23 | -1.67 |
|  | Month 24 | Aripiprazole - Paliperidone | 3.74 [-4.75, 12.23] | 0.39 | 1.35 |
|  |  |  |  |  |  |
| MoCA | Month 12 | Aripiprazole - TAU | 0.18 [-0.86, 1.21] | 0.74 | 0.08 |
|  | Month 12 | Paliperidone - TAU | -0.28 [-1.29, 0.73] | 0.59 | -0.13 |
|  | Month 12 | Aripiprazole - Paliperidone | 0.46 [-0.74, 1.66] | 0.46 | 0.21 |
|  | Month 24 | Aripiprazole - TAU | -0.49 [-1.65, 0.66] | 0.4 | -0.23 |
|  | Month 24 | Paliperidone - TAU | -2.06 [-3.28, -0.85] | 0 | -0.97 |
|  | Month 24 | Aripiprazole - Paliperidone | 1.57 [0.17, 2.97] | 0.03 | 0.74 |
|  |  |  |  |  |  |
| FAB | Month 12 | Aripiprazole - TAU | 0.63 [-0.48, 1.74] | 0.27 | 0.3 |
|  | Month 12 | Paliperidone - TAU | 0.4 [-0.67, 1.47] | 0.46 | 0.19 |
|  | Month 12 | Aripiprazole - Paliperidone | 0.23 [-1.06, 1.52] | 0.73 | 0.11 |
|  | Month 24 | Aripiprazole - TAU | 0.16 [-1.07, 1.39] | 0.8 | 0.08 |
|  | Month 24 | Paliperidone - TAU | 0.37 [-0.91, 1.65] | 0.57 | 0.18 |
|  | Month 24 | Aripiprazole - Paliperidone | -0.22 [-1.73, 1.29] | 0.78 | -0.1 |
|  |  |  |  |  |  |
| ASI |  |  |  |  |  |
| Medical status | Month 12 | Aripiprazole - TAU | 0.07 [-0.09, 0.22] | 0.41 | 0.09 |
|  | Month 12 | Paliperidone - TAU | -0.01 [-0.16, 0.14] | 0.87 | -0.02 |
|  | Month 12 | Aripiprazole - Paliperidone | 0.08 [-0.1, 0.26] | 0.4 | 0.11 |
|  | Month 24 | Aripiprazole - TAU | 0.12 [-0.06, 0.3] | 0.2 | 0.16 |
|  | Month 24 | Paliperidone - TAU | -0.13 [-0.32, 0.05] | 0.16 | -0.18 |
|  | Month 24 | Aripiprazole - Paliperidone | 0.25 [0.03, 0.47] | 0.03 | 0.34 |
| Employment/ Support Status | Month 12 | Aripiprazole - TAU | 0.03 [-0.06, 0.13] | 0.51 | NA |
|  | Month 12 | Paliperidone - TAU | 0.08 [-0.01, 0.17] | 0.1 | NA |
|  | Month 12 | Aripiprazole - Paliperidone | -0.04 [-0.15, 0.06] | 0.43 | NA |
|  | Month 24 | Aripiprazole - TAU | 0.02 [-0.08, 0.12] | 0.72 | NA |
|  | Month 24 | Paliperidone - TAU | 0.01 [-0.1, 0.11] | 0.88 | NA |
|  | Month 24 | Aripiprazole - Paliperidone | 0.01 [-0.11, 0.13] | 0.86 | NA |
|  |  |  |  |  |  |
| Alcohol | Month 12 | Aripiprazole - TAU | 0.0 [-0.06, 0.06] | 0.98 | NA |
|  | Month 12 | Paliperidone - TAU | 0.06 [0.0, 0.11] | 0.04 | NA |
|  | Month 12 | Aripiprazole - Paliperidone | -0.06 [-0.12, 0.01] | 0.09 | NA |
|  | Month 24 | Aripiprazole - TAU | -0.02 [-0.12, 0.08] | 0.74 | NA |
|  | Month 24 | Paliperidone - TAU | 0.05 [-0.06, 0.16] | 0.36 | NA |
|  | Month 24 | Aripiprazole - Paliperidone | -0.07 [-0.19, 0.06] | 0.29 | NA |
|  |  |  |  |  |  |
| Drugs | Month 12 | Aripiprazole - TAU | 0.01 [-0.04, 0.06] | 0.71 | NA |
|  | Month 12 | Paliperidone - TAU | 0.04 [-0.01, 0.09] | 0.15 | NA |
|  | Month 12 | Aripiprazole - Paliperidone | -0.03 [-0.09, 0.03] | 0.37 | NA |
|  | Month 24 | Aripiprazole - TAU | 0.01 [-0.03, 0.06] | 0.56 | NA |
|  | Month 24 | Paliperidone - TAU | 0.05 [-0.0, 0.1] | 0.07 | NA |
|  | Month 24 | Aripiprazole - Paliperidone | -0.03 [-0.09, 0.03] | 0.27 | NA |
|  |  |  |  |  |  |
| Legal status | Month 12 | Aripiprazole - TAU | 0.0 [-0.06, 0.07] | 0.92 | NA |
|  | Month 12 | Paliperidone - TAU | -0.02 [-0.08, 0.05] | 0.62 | NA |
|  | Month 12 | Aripiprazole - Paliperidone | 0.02 [-0.06, 0.1] | 0.62 | NA |
|  | Month 24 | Aripiprazole - TAU | -0.07 [-0.15, 0.01] | 0.09 | NA |
|  | Month 24 | Paliperidone - TAU | -0.09 [-0.17, -0.0] | 0.05 | NA |
|  | Month 24 | Aripiprazole - Paliperidone | 0.02 [-0.08, 0.11] | 0.74 | NA |
|  |  |  |  |  |  |
| Family/ Social relationships | Month 12 | Aripiprazole - TAU | 0.01 [-0.07, 0.09] | 0.81 | NA |
|  | Month 12 | Paliperidone - TAU | -0.07 [-0.14, 0.01] | 0.08 | NA |
|  | Month 12 | Aripiprazole - Paliperidone | 0.08 [-0.01, 0.17] | 0.1 | NA |
|  | Month 24 | Aripiprazole - TAU | 0.06 [-0.0, 0.12] | 0.07 | NA |
|  | Month 24 | Paliperidone - TAU | -0.03 [-0.09, 0.04] | 0.38 | NA |
|  | Month 24 | Aripiprazole - Paliperidone | 0.09 [0.01, 0.16] | 0.03 | NA |
|  |  |  |  |  |  |
| Psychiatric status | Month 12 | Aripiprazole - TAU | -0.06 [-0.16, 0.03] | 0.2 | NA |
|  | Month 12 | Paliperidone - TAU | -0.01 [-0.1, 0.08] | 0.86 | NA |
|  | Month 12 | Aripiprazole - Paliperidone | -0.05 [-0.16, 0.05] | 0.33 | NA |
|  | Month 24 | Aripiprazole - TAU | 0.01 [-0.09, 0.1] | 0.91 | NA |
|  | Month 24 | Paliperidone - TAU | 0.02 [-0.08, 0.11] | 0.74 | NA |
|  | Month 24 | Aripiprazole - Paliperidone | -0.01 [-0.12, 0.1] | 0.85 | NA |
|  |  |  |  |  |  |
| Frequency of drug use | Month 12 | Aripiprazole - TAU | -0.89 [-3.92, 2.13] | 0.56 | -0.35 |
|  | Month 12 | Paliperidone - TAU | 1.04 [-1.92, 4.01] | 0.49 | 0.4 |
|  | Month 12 | Aripiprazole - Paliperidone | -1.94 [-5.45, 1.57] | 0.28 | -0.75 |
|  | Month 24 | Aripiprazole - TAU | 0.8 [-3.0, 4.61] | 0.68 | 0.31 |
|  | Month 24 | Paliperidone - TAU | 0.05 [-3.9, 4.01] | 0.98 | 0.02 |
|  | Month 24 | Aripiprazole - Paliperidone | 0.75 [-3.83, 5.33] | 0.75 | 0.29 |

*Note*. NA: the estimated model-based variance used for standardization was numerically unstable or near zero, thus standardized effect sizes are not computable; ASI: Addiction Severity Index, BPRS-24: Brief Psychiatric Rating Scale–24 items, CGI: Clinical Global Impression, DSM-5: Diagnostic and Statistical Manual of Mental Disorders, Fifth Edition, FAB: Frontal Assessment Battery, GASS: Glasgow Antipsychotic Side-effect Scale, MoCA: Montreal Cognitive Assessment, StUD: Stimulant Use Disorder, SDS: Severity of Dependence Scale, SOCRATES-D: Stages of Change Readiness and Treatment Eagerness Scale-Drug.

**Supplementary Table 11.** Summary of drug urine test results for non-stimulants in the intention-to-treat samples (n=165) at baseline, 12-month primary and 24-month secondary endpoints.

| Month | Substance | Group | Positive test (%) | Chi-square | p-value |
| --- | --- | --- | --- | --- | --- |
| 0 | Cannabis | Aripiprazole | 3/39 (7.69) | 0.42 | 0.81 |
|  |  | Paliperidone | 2/38 (5.26) |  |  |
|  |  | TAU | 4/83 (4.82) |  |  |
| 12 |  | Aripiprazole | 1/23 (4.35) | 0.07 | 0.97 |
|  |  | Paliperidone | 1/27 (3.70) |  |  |
|  |  | TAU | 2/63 (3.17) |  |  |
| 24 |  | Aripiprazole | 0/19 (0) | 2.38 | 0.30 |
|  |  | Paliperidone | 0/17 (0) |  |  |
|  |  | TAU | 3/47 (6.38) |  |  |
|  |  |  |  |  |  |
| 0 | Ketamine | Aripiprazole | 2/39 (5.13) | 0.34 | 0.84 |
|  |  | Paliperidone | 1/38 (2.63) |  |  |
|  |  | TAU | 3/83 (3.61) |  |  |
| 12 |  | Aripiprazole | 2/23 (8.70) | 0.03 | 0.99 |
|  |  | Paliperidone | 2/27 (7.41) |  |  |
|  |  | TAU | 5/63 (7.94) |  |  |
| 24 |  | Aripiprazole | 0/19 (0) | 5.53 | 0.06 |
|  |  | Paliperidone | 3/17 (17.65) |  |  |
|  |  | TAU | 2/47 (4.26) |  |  |
|  |  |  |  |  |  |
| 0 | Opioids | Aripiprazole | 2/39 (5.13) | 5.04 | 0.08 |
|  |  | Paliperidone | 5/38 (13.16) |  |  |
|  |  | TAU | 17/83 (20.48) |  |  |
| 12 |  | Aripiprazole | 3/23 (13.04) | 0.17 | 0.92 |
|  |  | Paliperidone | 3/27 (11.11) |  |  |
|  |  | TAU | 9/63 (14.29) |  |  |
| 24 |  | Aripiprazole | 2/19 (10.53) | 1.20 | 0.55 |
|  |  | Paliperidone | 4/17 (23.53) |  |  |
|  |  | TAU | 7/47 (14.89) |  |  |
|  |  |  |  |  |  |
| 0 | Sedatives | Aripiprazole | 2/39 (5.13) | 1.13 | 0.57 |
|  |  | Paliperidone | 3/38 (7.89) |  |  |
|  |  | TAU | 9/83 (10.84) |  |  |
| 12 |  | Aripiprazole | 1/23 (4.35) | 0.05 | 0.98 |
|  |  | Paliperidone | 1/27 (3.70) |  |  |
|  |  | TAU | 3/63 (4.76) |  |  |
| 24 |  | Aripiprazole | 0/19 (0) | 1.57 | 0.46 |
|  |  | Paliperidone | 0/17 (0) |  |  |
|  |  | TAU | 2/47 (4.26) |  |  |

Note. Not all randomized participants provided their urine samples at follow-up timepoints. Numerator represents the number of positive urine test results for the substance; denominator represents the total number of urine sample collected. TAU: treatment-as-usual. Sedatives included barbiturates and benzodiazepines. Opioids included heroin, morphine, methadone and codeine.

**Supplementary Table 12.** Pairwise comparisons of all primary and secondary outcomes in the sensitivity analysis (n = 137) with between the three treatment groups at the primary and secondary endpoints.

| Outcomes | Endpoints | Contrast | Estimate (95% CI) | p-value | Cohens d_ |
| --- | --- | --- | --- | --- | --- |
| CGI-S | Month 12 | Aripiprazole - TaU | -0.4 [-0.89, 0.1] | 0.12 | -0.23 |
|  | Month 12 | Paliperidone - TaU | -0.2 [-0.74, 0.34] | 0.48 | -0.11 |
|  | Month 12 | Aripiprazole - Paliperidone | -0.2 [-0.82, 0.42] | 0.53 | -0.12 |
|  | Month 24 | Aripiprazole - TaU | -0.67 [-1.3, -0.04] | **0.04** | -0.39 |
|  | Month 24 | Paliperidone - TaU | 0.21 [-0.5, 0.91] | 0.57 | 0.12 |
|  | Month 24 | Aripiprazole - Paliperidone | -0.88 [-1.68, -0.07] | **0.04** | -0.51 |
| CGI-I | Month 12 | Aripiprazole - TaU | -0.6 [-1.23, 0.04] | 0.07 | -0.32 |
|  | Month 12 | Paliperidone - TaU | -0.64 [-1.3, 0.01] | 0.06 | -0.35 |
|  | Month 12 | Aripiprazole - Paliperidone | 0.05 [-0.71, 0.81] | 0.9 | 0.03 |
|  | Month 24 | Aripiprazole - TaU | -0.8 [-1.52, -0.08] | **0.03** | -0.44 |
|  | Month 24 | Paliperidone - TaU | 0.95 [0.15, 1.75] | **0.02** | 0.52 |
|  | Month 24 | Aripiprazole - Paliperidone | -1.75 [-2.65, -0.84] | **0** | -0.95 |
| CGI-E | Month 12 | Aripiprazole - TaU | -4.2 [-6.69, -1.7] | **0** | -1.73 |
|  | Month 12 | Paliperidone - TaU | -2.45 [-4.97, 0.06] | 0.06 | -1.01 |
|  | Month 12 | Aripiprazole - Paliperidone | -1.74 [-4.65, 1.17] | 0.24 | -0.72 |
|  | Month 24 | Aripiprazole - TaU | -5.15 [-7.69, -2.61] | **0** | -2.13 |
|  | Month 24 | Paliperidone - TaU | -1.8 [-4.5, 0.91] | 0.2 | -0.74 |
|  | Month 24 | Aripiprazole - Paliperidone | -3.36 [-6.46, -0.25] | **0.04** | -1.39 |
| BPRS-24 | Month 12 | Aripiprazole - TaU | -0.75 [-3.56, 2.05] | 0.6 | -0.3 |
|  | Month 12 | Paliperidone - TaU | -0.24 [-3.21, 2.74] | 0.88 | -0.1 |
|  | Month 12 | Aripiprazole - Paliperidone | -0.52 [-3.91, 2.88] | 0.77 | -0.21 |
|  | Month 24 | Aripiprazole - TaU | -0.86 [-3.95, 2.24] | 0.59 | -0.34 |
|  | Month 24 | Paliperidone - TaU | 1.97 [-1.47, 5.41] | 0.27 | 0.79 |
|  | Month 24 | Aripiprazole - Paliperidone | -2.83 [-6.73, 1.08] | 0.16 | -1.14 |
| GASS | Month 12 | Aripiprazole - TaU | -0.58 [-4.57, 3.42] | 0.78 | -0.22 |
|  | Month 12 | Paliperidone - TaU | 2.43 [-1.9, 6.75] | 0.27 | 0.92 |
|  | Month 12 | Aripiprazole - Paliperidone | -3.0 [-7.92, 1.91] | 0.23 | -1.13 |
|  | Month 24 | Aripiprazole - TaU | 4.13 [-0.18, 8.45] | 0.06 | 1.56 |
|  | Month 24 | Paliperidone - TaU | 1.91 [-2.95, 6.76] | 0.44 | 0.72 |
|  | Month 24 | Aripiprazole - Paliperidone | 2.23 [-3.24, 7.69] | 0.43 | 0.84 |
| Severity of DSM-5 StUD | Month 12 | Aripiprazole - TaU | 0.13 [-1.11, 1.38] | 0.83 | 0.06 |
|  | Month 12 | Paliperidone - TaU | 0.24 [-1.11, 1.6] | 0.72 | 0.11 |
|  | Month 12 | Aripiprazole - Paliperidone | -0.11 [-1.68, 1.46] | 0.89 | -0.05 |
|  | Month 24 | Aripiprazole - TaU | -0.75 [-2.39, 0.89] | 0.37 | -0.35 |
|  | Month 24 | Paliperidone - TaU | 0.19 [-1.79, 2.17] | 0.85 | 0.09 |
|  | Month 24 | Aripiprazole - Paliperidone | -0.94 [-3.18, 1.3] | 0.41 | -0.44 |
| SDS | Month 12 | Aripiprazole - TaU | 0.06 [-1.25, 1.37] | 0.93 | 0.03 |
|  | Month 12 | Paliperidone - TaU | 0.86 [-0.5, 2.21] | 0.22 | 0.39 |
|  | Month 12 | Aripiprazole - Paliperidone | -0.8 [-2.37, 0.77] | 0.32 | -0.36 |
|  | Month 24 | Aripiprazole - TaU | 1.31 [-0.47, 3.09] | 0.15 | 0.59 |
|  |  |  |  |  |  |
|  | Month 24 | Paliperidone - TaU | 1.31 [-0.62, 3.24] | 0.19 | 0.59 |
|  | Month 24 | Aripiprazole - Paliperidone | 0.0 [-2.15, 2.16] | 1 | 0 |
| SOCRATES-D | Month 12 | Aripiprazole - TaU | 2.68 [-1.33, 6.69] | 0.19 | 0.97 |
|  | Month 12 | Paliperidone - TaU | -1.09 [-5.37, 3.18] | 0.62 | -0.4 |
|  | Month 12 | Aripiprazole - Paliperidone | 3.77 [-0.95, 8.5] | 0.12 | 1.37 |
|  | Month 24 | Aripiprazole - TaU | 0.14 [-6.83, 7.11] | 0.97 | 0.05 |
|  | Month 24 | Paliperidone - TaU | -2.69 [-10.58, 5.2] | 0.51 | -0.98 |
|  | Month 24 | Aripiprazole - Paliperidone | 2.83 [-5.62, 11.28] | 0.51 | 1.03 |
| MoCA | Month 12 | Aripiprazole - TaU | 0.26 [-0.79, 1.3] | 0.63 | 0.12 |
|  | Month 12 | Paliperidone - TaU | 0.02 [-1.12, 1.16] | 0.98 | 0.01 |
|  | Month 12 | Aripiprazole - Paliperidone | 0.24 [-1.06, 1.53] | 0.72 | 0.11 |
|  | Month 24 | Aripiprazole - TaU | -0.2 [-1.38, 0.98] | 0.74 | -0.09 |
|  | Month 24 | Paliperidone - TaU | -1.83 [-3.17, -0.48] | **0.01** | -0.87 |
|  | Month 24 | Aripiprazole - Paliperidone | 1.63 [0.13, 3.13] | **0.04** | 0.78 |
| FAB | Month 12 | Aripiprazole - TaU | 0.51 [-0.59, 1.61] | 0.37 | 0.24 |
|  | Month 12 | Paliperidone - TaU | 1.01 [-0.19, 2.2] | 0.1 | 0.48 |
|  | Month 12 | Aripiprazole - Paliperidone | -0.5 [-1.87, 0.87] | 0.48 | -0.24 |
|  | Month 24 | Aripiprazole - TaU | 0.03 [-1.3, 1.36] | 0.97 | 0.01 |
|  | Month 24 | Paliperidone - TaU | 0.08 [-1.43, 1.59] | 0.92 | 0.04 |
|  | Month 24 | Aripiprazole - Paliperidone | -0.05 [-1.77, 1.66] | 0.95 | -0.03 |
| ASI-Medical | Month 12 | Aripiprazole - TaU | 0.09 [-0.07, 0.26] | 0.27 | 0.13 |
|  | Month 12 | Paliperidone - TaU | 0.01 [-0.17, 0.19] | 0.94 | 0.01 |
|  | Month 12 | Aripiprazole - Paliperidone | 0.09 [-0.12, 0.29] | 0.42 | 0.12 |
|  | Month 24 | Aripiprazole - TaU | 0.06 [-0.14, 0.25] | 0.58 | 0.08 |
|  | Month 24 | Paliperidone - TaU | -0.17 [-0.39, 0.05] | 0.14 | -0.24 |
|  | Month 24 | Aripiprazole - Paliperidone | 0.23 [-0.04, 0.49] | 0.1 | 0.31 |
| ASI-Employment | Month 12 | Aripiprazole - TaU | 0.03 [-0.07, 0.13] | 0.6 | NA |
|  | Month 12 | Paliperidone - TaU | 0.07 [-0.04, 0.18] | 0.19 | NA |
|  | Month 12 | Aripiprazole - Paliperidone | -0.04 [-0.17, 0.08] | 0.48 | NA |
|  | Month 24 | Aripiprazole - TaU | -0.0 [-0.11, 0.1] | 0.96 | NA |
|  | Month 24 | Paliperidone - TaU | 0.03 [-0.09, 0.14] | 0.65 | NA |
|  | Month 24 | Aripiprazole - Paliperidone | -0.03 [-0.16, 0.1] | 0.67 | NA |
| ASI-Alcohol | Month 12 | Aripiprazole - TaU | -0.01 [-0.06, 0.05] | 0.84 | NA |
|  | Month 12 | Paliperidone - TaU | 0.02 [-0.04, 0.08] | 0.43 | NA |
|  | Month 12 | Aripiprazole - Paliperidone | -0.03 [-0.1, 0.04] | 0.39 | NA |
|  | Month 24 | Aripiprazole - TaU | -0.02 [-0.1, 0.06] | 0.66 | NA |
|  | Month 24 | Paliperidone - TaU | 0.01 [-0.08, 0.11] | 0.77 | NA |
|  | Month 24 | Aripiprazole - Paliperidone | -0.03 [-0.14, 0.08] | 0.55 | NA |
| ASI-Drugs | Month 12 | Aripiprazole - TaU | 0.01 [-0.05, 0.07] | 0.73 | NA |
|  | Month 12 | Paliperidone - TaU | 0.06 [-0.01, 0.12] | 0.07 | NA |
|  | Month 12 | Aripiprazole - Paliperidone | -0.05 [-0.12, 0.02] | 0.2 | NA |
|  | Month 24 | Aripiprazole - TaU | 0.02 [-0.03, 0.07] | 0.37 | NA |
|  | Month 24 | Paliperidone - TaU | 0.07 [0.02, 0.13] | 0.01 | NA |
|  | Month 24 | Aripiprazole - Paliperidone | -0.05 [-0.11, 0.01] | 0.12 | NA |
| ASI-Legal status | Month 12 | Aripiprazole - TaU | 0.02 [-0.05, 0.09] | 0.66 | NA |
|  | Month 12 | Paliperidone - TaU | 0.0 [-0.07, 0.08] | 0.91 | NA |
|  | Month 12 | Aripiprazole - Paliperidone | 0.01 [-0.08, 0.1] | 0.8 | NA |
|  | Month 24 | Aripiprazole - TaU | -0.06 [-0.14, 0.03] | 0.18 | NA |
|  | Month 24 | Paliperidone - TaU | -0.08 [-0.18, 0.02] | 0.11 | NA |
|  | Month 24 | Aripiprazole - Paliperidone | 0.02 [-0.09, 0.13] | 0.72 | NA |
| ASI-Family/ Social relationships | Month 12 | Aripiprazole - TaU | 0.01 [-0.07, 0.1] | 0.77 | NA |
|  | Month 12 | Paliperidone - TaU | -0.07 [-0.16, 0.02] | 0.13 | NA |
|  | Month 12 | Aripiprazole - Paliperidone | 0.08 [-0.02, 0.19] | 0.13 | NA |
|  | Month 24 | Aripiprazole - TaU | 0.07 [0.0, 0.14] | 0.04 | NA |
|  | Month 24 | Paliperidone - TaU | -0.03 [-0.11, 0.05] | 0.44 | NA |
|  | Month 24 | Aripiprazole - Paliperidone | 0.1 [0.01, 0.19] | **0.03** | NA |
| ASI-Psychiatric status | Month 12 | Aripiprazole - TaU | -0.03 [-0.13, 0.07] | 0.51 | NA |
|  | Month 12 | Paliperidone - TaU | 0.06 [-0.05, 0.16] | 0.28 | NA |
|  | Month 12 | Aripiprazole - Paliperidone | -0.09 [-0.22, 0.03] | 0.15 | NA |
|  | Month 24 | Aripiprazole - TaU | 0.02 [-0.08, 0.12] | 0.67 | NA |
|  | Month 24 | Paliperidone - TaU | 0.07 [-0.04, 0.18] | 0.23 | NA |
|  | Month 24 | Aripiprazole - Paliperidone | -0.05 [-0.17, 0.08] | 0.49 | NA |
| ASI-Frequency of drug use | Month 12 | Aripiprazole - TaU | -1.18 [-4.45, 2.09] | 0.48 | -0.45 |
|  | Month 12 | Paliperidone - TaU | 2.37 [-1.24, 5.98] | 0.2 | 0.91 |
|  | Month 12 | Aripiprazole - Paliperidone | -3.55 [-7.64, 0.54] | 0.09 | -1.37 |
|  | Month 24 | Aripiprazole - TaU | 0.31 [-3.79, 4.4] | 0.88 | 0.12 |
|  | Month 24 | Paliperidone - TaU | -0.44 [-5.11, 4.24] | 0.86 | -0.17 |
|  | Month 24 | Aripiprazole - Paliperidone | 0.74 [-4.49, 5.97] | 0.78 | 0.29 |

*Note*. NA: the estimated model-based variance used for standardization was numerically unstable or near zero, thus standardized effect sizes are not computable. ASI: Addiction Severity Index, BPRS-24: Brief Psychiatric Rating Scale–24 items, CGI: Clinical Global Impression, DSM-5: Diagnostic and Statistical Manual of Mental Disorders, Fifth Edition, FAB: Frontal Assessment Battery, GASS: Glasgow Antipsychotic Side-effect Scale, MoCA: Montreal Cognitive Assessment, StUD: Stimulant Use Disorder, SDS: Severity of Dependence Scale, SOCRATES-D: Stages of Change Readiness and Treatment Eagerness Scale-Drug.

**Supplementary Figure 1.** Estimated changes from baseline in primary and secondary outcomes over the two-year study period.

*Note*. ASI: Addiction Severity Index, BPRS-24: Brief Psychiatric Rating Scale–24 item, CGI: Clinical Global Impression, FAB: Frontal Assessment Battery, GASS: Glasgow Antipsychotic Side-effect Scale, MoCA: Montreal Cognitive Assessment, SCID-5-CV: Structured Clinical Interview for DSM-5 Disorders–Clinician Version, StIP: Stimulant-induced Psychosis, StUD: Stimulant Use Disorder, SDS: Severity of Dependence Scale, SOCRATES-D: Stages of Change Readiness and Treatment Eagerness Scale–Drug, TAU: Treatment-as-usual. Error bars represent ±1 standard error.

**Supplementary Figure 2**. Intention-to-treat samples ( n = 165) vs sensitivity analysis samples (n = 137) on primary and secondary outcomes over the two-year study period. Note: A: aripiprazole; P: paliperidone; T: treatment-as-usual.
